# To Switch or Not to Switch: Role of Cognitive Control in Working Memory Training in Older Adults

**DOI:** 10.3389/fpsyg.2016.00230

**Published:** 2016-03-02

**Authors:** Chandramallika Basak, Margaret A. O’Connell

**Affiliations:** Center for Vital Longevity, School of Behavioral and Brain Sciences, University of Texas at Dallas, RichardsonTX, USA

**Keywords:** working memory training, cognitive control, healthy aging, strategies of training, individual differences

## Abstract

It is currently not known what are the best working memory training strategies to offset the age-related declines in fluid cognitive abilities. In this randomized clinical double-blind trial, older adults were randomly assigned to one of two types of working memory training – one group was trained on a predictable memory updating task (PT) and another group was trained on a novel, unpredictable memory updating task (UT). Unpredictable memory updating, compared to predictable, requires greater demands on cognitive control (Basak and Verhaeghen, 2011a). Therefore, the current study allowed us to evaluate the role of cognitive control in working memory training. All participants were assessed on a set of near and far transfer tasks at three different testing sessions – before training, immediately after the training, and 1.5 months after completing the training. Additionally, individual learning rates for a comparison working memory task (performed by both groups) and the trained task were computed. Training on unpredictable memory updating, compared to predictable, significantly enhanced performance on a measure of episodic memory, immediately after the training. Moreover, individuals with faster learning rates showed greater gains in this episodic memory task and another new working memory task; this effect was specific to UT. We propose that the unpredictable memory updating training, compared to predictable memory updating training, may a better strategy to improve selective cognitive abilities in older adults, and future studies could further investigate the role of cognitive control in working memory training.

## Introduction

In order to maintain quality of life until late adulthood and decrease the health burden of a rapidly aging society, it is important that we develop an understanding of the principles of cognitive optimization, because gains in longevity have not been matched by maintenance of cognitive function into very old age. In particular, fluid cognition declines rapidly with age, particularly after 60 years, and includes abilities such as episodic memory, reasoning, and multi-tasking (Park and Bischof, 2010; Stine-Morrow and Basak, 2011). A plausible reason for impairments in these cognitive abilities with age is the disruption of the fronto-parietal brain networks that underlie working memory and cognitive control (Park and Reuter-Lorenz, 2009; Raz et al., 2010). One proposed principle of cognitive optimization is the enhancement of cognitive control in working memory, particularly in older adults (Basak and Zelinski, 2013).

Both cognitive control and working memory have been argued to be the underlying “core” components of fluid cognition (Stine-Morrow and Basak, 2011). Working memory, the ability to concurrently store and actively transform information (e.g., Mayr et al., 1996), is related to many complex cognitive skills, e.g., reasoning (Kyllonen and Christal, 1990). It also underlies many age-related deficits in fluid cognition, including episodic memory (Verhaeghen and Salthouse, 1997; Verhaeghen et al., 2005; Lewis and Zelinski, 2010). Moreover, significant and early declines of verbal episodic memory have long been considered to be the best cognitive marker of the earliest stages of Alzheimer’s disease (Dubois et al., 2007). Therefore, training cognitively healthy older adults in these “core” cognitive components may not merely improve their fluid cognitive abilities, but can also potentially delay the onset of memory-related disorders, such as Alzheimer’s disease.

The primary aim of the current study was to evaluate two different strategies to optimize cognition in older adults over a short period time by using theory-driven, simple cognitive training protocols. An understanding of the role of cognitive control in working memory, including the variations in the retrieval-related temporal dynamics and its adaptability with extensive practice, can inform us about the best strategies to use to enhance cognitive vitality into late adulthood. Such informed principles of cognitive optimization may potentially delay the onset of pathological memory-related disorders in the healthy aging population, and in turn, decrease the medical-care burden of a rapidly aging society.

### Predictability of Focus Switching and Aging in Working Memory

Cowan’s hierarchical model of working memory (Cowan, 1988, 2001) posited a two-tier hierarchy based on the accessibility of information via a zone of immediate access, labeled the *focus of attention* (FoA), and a larger activated portion of long-term memory (LTM), where the items are stored in a readily available but not in an immediately accessible state. One of the most intriguing findings in cognitive psychology has been the limited capacity of the FoA. For tasks requiring serial attention processes, e.g., the continuous memory updating paradigms (McElree, 2001; Oberauer, 2002, 2006; Verhaeghen et al., 2004; Verhaeghen and Basak, 2005; Vaughan et al., 2008; Basak and Verhaeghen, 2011a,b), the capacity of FoA has been limited to just one item. If more than one information unit was to be processed, the other information units were temporarily stored in the outer store, while the current information in the FoA was updated. To process an item stored in the outer store, a retrieval operation was required that shifted the item from the outer store into the FoA (*focus switch*). This focus switch process increased the retrieval latency of that information (Verhaeghen and Basak, 2005). Therefore, measurement of the capacity of the FoA has typically involved the assessment of the *focus switch costs*, which is considered to be a measure of cognitive control (Garavan, 1998; Verhaeghen and Basak, 2005). Retrieval dynamics of the zone outside the FoA have been disputed between two prominent theories. One theory has proposed that these retrieval dynamics, viz., focus switch costs, are constant (McElree, 2001), whereas the other theory has argued that they increase as a function of the number of items in the outer store (Oberauer, 2002). Due to this disagreement between the two theories, we shall here refer to the zone outside the FoA as the “*outer store*” (Verhaeghen et al., 2004; Verhaeghen and Basak, 2005).

**FIGURE 1:**
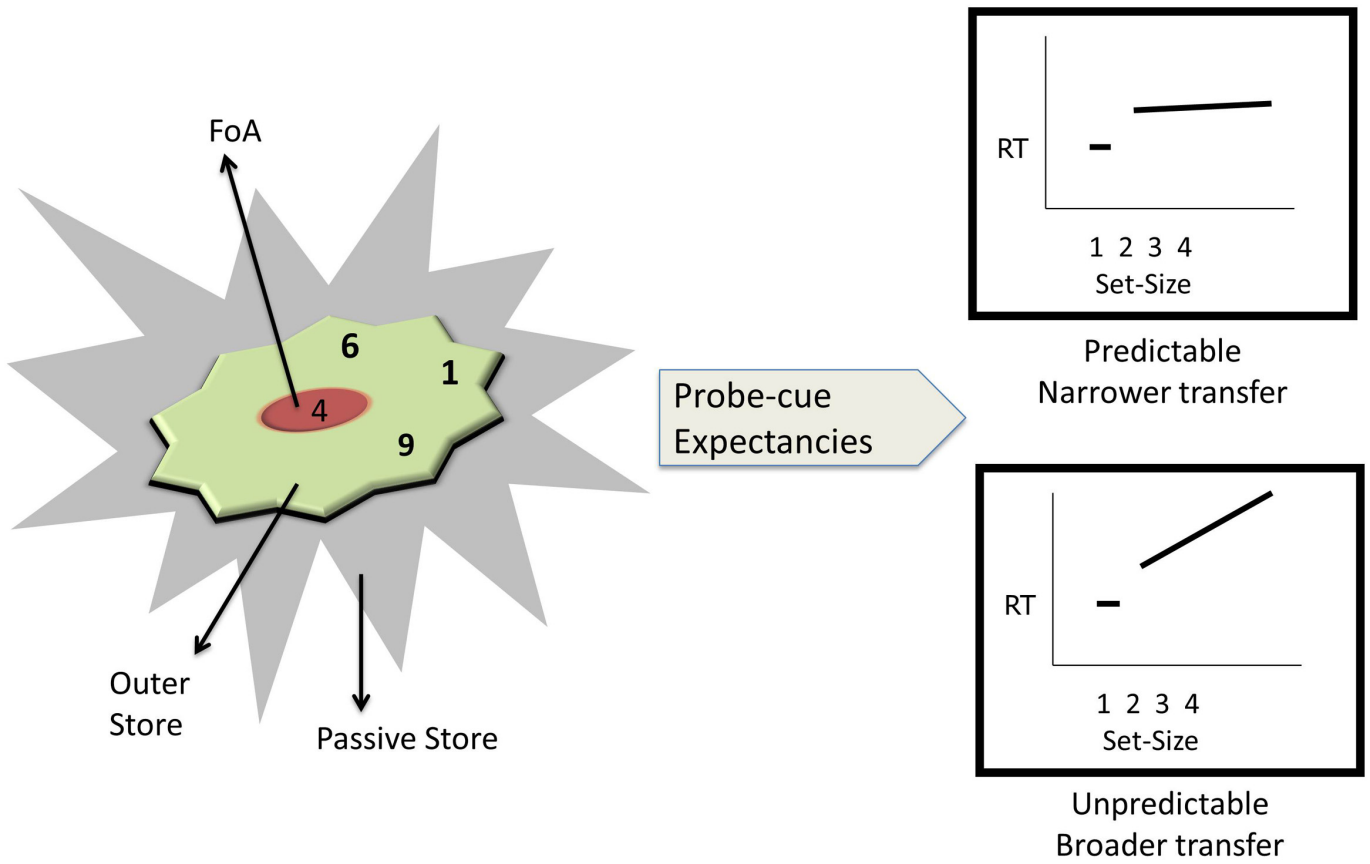
**The Theory of Working Memory Adaptability (ToWMA) posits three-tiered architecture for representations of information in human working memory.** It consists of a passive store, which is firewalled from the two active stores, viz., the outer store and a limited focus of attention. The two active stores together are hypothesized to have a capacity of 4 ± 1 information units, with the retrieval dynamics in the outer store depending on the probe-cue expectancies. When the probe-cue expectancies are predictable, the focus switch costs are constant as a function of set-size in the outer store (Top Right). When the probe-cue expectancies are unpredictable, the focus switch costs are hypothesized to increase with increases in memory set-size (Bottom Right), and extensive practice on such paradigms is hypothesized to engender broader transfer to fluid cognition.

The current study was guided by a previously published hierarchical theory of working memory (Verhaeghen et al., 2004; Verhaeghen and Basak, 2005; Basak, 2006; Vaughan et al., 2008; Basak and Verhaeghen, 2011a,b; Basak and Zelinski, 2013), henceforth referred to as the Theory of Working Memory Adaptability (ToWMA; see **Figure 1**). This theory is both significant and novel in integrating three different families of results regarding the hierarchies of working memory (Cowan, 1988; McElree, 2001; Oberauer, 2002, 2006; Verhaeghen et al., 2004; Verhaeghen and Basak, 2005; Basak, 2006; Vaughan et al., 2008; Basak and Verhaeghen, 2011a,b) by accounting for the probe-cue expectancy that is missing from the previous hierarchical models. Importantly, this theory makes specific predictions regarding the retrieval-related temporal dynamics in the outer store, the change in these dynamics over time, and the best strategies to improve a variety of untrained fluid cognitive skills in both younger and older adults.

According to ToWMA, the three-tier working memory architecture constitutes of an inner *focus of attention* of one information unit, an *outer store* where information that needs active manipulation and subsequent updating is maintained, and a *passive store* where information that does not need any updating is held for subsequent retrieval (Basak and Zelinski, 2013). ToWMA also posits that the passive store is firewalled against the active zones, viz., FoA and outer store. ToWMA differs from other models regarding the functions of FoA as well as the retrieval dynamics of the outer store. According to ToWMA, focus of attention has three functions – directing attention to the relevant information, retrieving the information, and subsequently updating the information (Basak and Verhaeghen, 2011a). The predictability of probe-cue expectancy has been hypothesized to affect FoA’s ability to direct attention to the relevant target. This, in turn, can affect the focus switch cost of information units in the outer store.

As mentioned before, there is an ongoing debate regarding the retrieval dynamics of information units in the outer store (McElree, 2001; Oberauer, 2002). According to Oberauer (2002), the focus switch cost increases as a function of set size. This is supported by models of serial processing, which posit that when searching for a specific item from a set of multiple items held in memory, response latency increases as a function of the number of items in the memory set. This suggests that the items in the memory set are examined individually until the target item is found. However, such increasing focus switch costs have not been replicated in other studies (McElree, 2001; Verhaeghen and Basak, 2005; Vaughan et al., 2008).

According to ToWMA, increases (or lack of it) in the focus-switch costs are hypothesized to be related to probe-cue expectancies. Unpredictable, compared to predictable, probe-cue expectancies engendered greater demands on cognitive control, indexed by the focus switch costs (Basak and Verhaeghen, 2011b). For example, in the N-back paradigms (McElree, 2001; Verhaeghen and Basak, 2005; Vaughan et al., 2008), where the probe-cue was always preceded by the same N positions, the expectancy was fixed or predictable. This predictable expectancy allowed the focus to be directed to the relevant target without much overhead cost of search or interference from other competing cues. This resulted in a constant focus switch cost from *N* = 2 to 5 (Verhaeghen and Basak, 2005; see top right of **Figure 1**). This pattern of constant focus switch cost for items in the outer store was unchanged even when the task difficulty was increased (Vaughan et al., 2008). Yet, in the unpredictable N-back task, where the position of the probe-cue within an N was random, the focus switch cost increased with N for N > 1 (Oberauer, 2002; Basak and Verhaeghen, 2011b; see bottom right of **Figure 1**). Such increase in latencies was considered to be an evidence of either a search process or a result of increased interference between competing active cues; alternative explanations, such as, lag of the last switch, were ruled out (Basak and Verhaeghen, 2011a). Also, the focus switch costs (*N* = 2 vs. 1) were of greater magnitude for the unpredictable, compared to the predictable, paradigms (Garavan, 1998; Verhaeghen and Basak, 2005; Basak and Verhaeghen, 2011a,b).

These results indicated that the unpredictable probe-cue expectancies engender greater cognitive control than the predictable probe-cue expectancies. Since age-related deficits are marked in cognitive control, ToWMA proposed that training in unpredictable versions of memory updating paradigms would engender greater transfer to tasks of fluid cognition in older adults, particularly those subserved by cognitive control (Basak and Zelinski, 2013).

### Working Memory Training and Aging

Results are mixed regarding transfer of N-back training to fluid abilities, where the training was typically adaptive (Jaeggi et al., 2008, 2010; Redick et al., 2013). Also, a lack of transfer in older adults, compared to younger adults, from working memory training has been attributed to age-related differences in patterns of brain activation for the trained and transfer tasks. In an fMRI study, younger adults showed overlap in the left striatum (thought to serve as a gate-keeping function for working memory) between the trained memory updating task and the N-back transfer task. In contrast, older adults showed no such overlap (Dahlin et al., 2008). In keeping with this argument, we have found that individual differences in striatal volume in younger (Erickson et al., 2010) and prefrontal cortex in older (Basak et al., 2011) are predictive of complex skill acquisition.

On the other hand a recent meta-analyses (Karbach and Verhaeghen, 2014), which evaluated the effects of task switch training versus working memory training on both younger and older adults, found that both types of training, particularly working memory, engendered transfer in both age groups. However, this meta-analysis did not evaluate the role of cognitive control in working memory training. It is plausible that any cognitive improvements to untrained tasks caused by working memory training resulted from training the ability to sustain and effectively control attention to the relevant information. Importantly, prior studies have only focused on immediate performance gains, compared to baseline performance, of the trained individuals; therefore, leaving the long-term benefits of working memory training in older adults unknown.

Although studies focusing on cognitive training in older individuals have failed to provide evidence for broad transfer to untrained cognitive skills (for a review, see Stine-Morrow and Basak, 2011), broader transfer has been observed in younger adults where cognitive control was trained through shifting task priorities in complex video games (Gopher et al., 1989; Kramer et al., 1995, 1999b; Boot et al., 2010; Lee et al., 2012a,b; Prakash et al., 2012; Voss et al., 2012). This approach, called variable priority training, compared to fixed priority training, relies more on the attentional control networks in the brain (Voss et al., 2012). Variable-, compared to fixed-, priority training in dual-task improved performance in a near transfer task (another dual-task) and two far transfer tasks (a running memory task requiring memory updating and a scheduling task requiring cognitive control) in both younger and older adults (Kramer et al., 1995). Variable-priority training also reduced age-related differences in the trained task and yielded greater long-term benefits after 6–8 weeks of completion of training (Kramer et al., 1999b). Moreover, fMRI studies in younger adults have shown differential increases in functional connectivity in the attentional control networks (e.g., fronto-parietal, fronto-executive) favoring variable training on both the trained task and a near transfer dual-task, suggesting that the variable-priority training taught generalizable cognitive control skills (Voss et al., 2012).

Unlike the fixed priority training, variable priority training is typically individualized adaptive as well as engages greater cognitive control by unpredictably shifting task priorities. Therefore, it is not possible to determine which of the two – greater cognitive control or individualized adaptive nature of the training – is the mechanism of transfer for variable-priority training. Moreover, new evidence suggests that working memory training may be more beneficial than dual-task training in inducing far transfer in older adults (Karbach and Verhaeghen, 2014). To date, studies conducted on variable priority training in older adults have used dual-task as the training paradigm, not working memory (Kramer et al., 1995; Erickson et al., 2007). Moreover, dual-task training in older adults has typically shown limited transfer, usually to another dual-task situation (Kramer et al., 1995; Bherer et al., 2005; Strobach et al., 2015).

On the other hand, working memory updating, compared to dual-tasking, has been argued to be more predictive of fluid intelligence (Friedman et al., 2006). Therefore, unpredictable memory updating training may have the potential to engender broader transfer to fluid cognitive skills than dual-task training. But we are not aware of any research that has explored the potential of unpredictable memory updating training – an approach where items to be updated are randomly retrieved. Existing research utilizing working memory updating tasks to train cognition have consistently employed predictable probe-cue paradigms, such as the N-back task. They have yielded mixed results regarding transfer of training (Jaeggi et al., 2008, 2010; Redick et al., 2013).

The current study was aimed to fulfill the afore-mentioned gap in the literature by explicitly assessing the prediction that unpredictable probe-cue memory updating training would be maximally effective for older adults in engendering broader transfer to fluid cognition. We manipulated probe-cue predictability – unpredictable vs. predictable – across the two types of training. Both trainings were not individualized adaptive. That is, all participants had to undergo all set-sizes in each training session, irrespective of their level of performance. The main research goal was to investigate the predictions of ToWMA, by comparing two different strategies of training working memory – one engendering greater demands on cognitive control than the other. According to ToWMA, the degree of probe-cue predictability affects the demands on cognitive control, such that, the unpredictable training (UT) paradigm required more cognitive control than the predictable training (PT) paradigm. Therefore, if cognitive control is the underlying mechanism of transfer in working memory training, then immediate post-testing gains (i.e., just after completion of the training) and, to a lesser extent, delayed post-testing gains (i.e., 1.5 months after completion of the training) were expected more for the UT in the tasks of fluid cognition.

## Materials and Methods

### Participants

Forty-six older adults, between 60 and 86 years old, were recruited for this randomized clinical double-blind experiment. Twenty-nine participants were female. All adults were right handed. Inclusion criteria included a minimum of a high school education, normal corrected vision of 20/30, and normal or prehypertension range of blood pressure (<140/90 mm HG) with or without medication. Exclusion criteria included color blindness, low familiarity of computer use, Mini Mental Status Examination (MMSE-2) < 25, and prior involvement in any type of cognitive training studies. Recruitment was conducted through flyers posted around the University of Texas at Dallas campus and surrounding businesses, and through advertisements posted on community newspapers. Participants were compensated at $10/h for their time and effort and were provided a bonus (Wave 1: $50; Wave 2 that included delayed post-testing: $100) for completing the multi-session experiment.

#### Power Analysis

A total sample size of 46 (combining across the two training groups) provides us with more than 90% power to detect a moderate effect size (f) of 0.25 at the 0.05 alpha level for the interaction term in a 2(Training_type) × 2(Session) ANOVA (http://www.psycho.uni-dusseldorf.de/abteilungen/gpower3). Therefore, data collection was stopped when 46 participants were recruited for the study.

**FIGURE 2:**
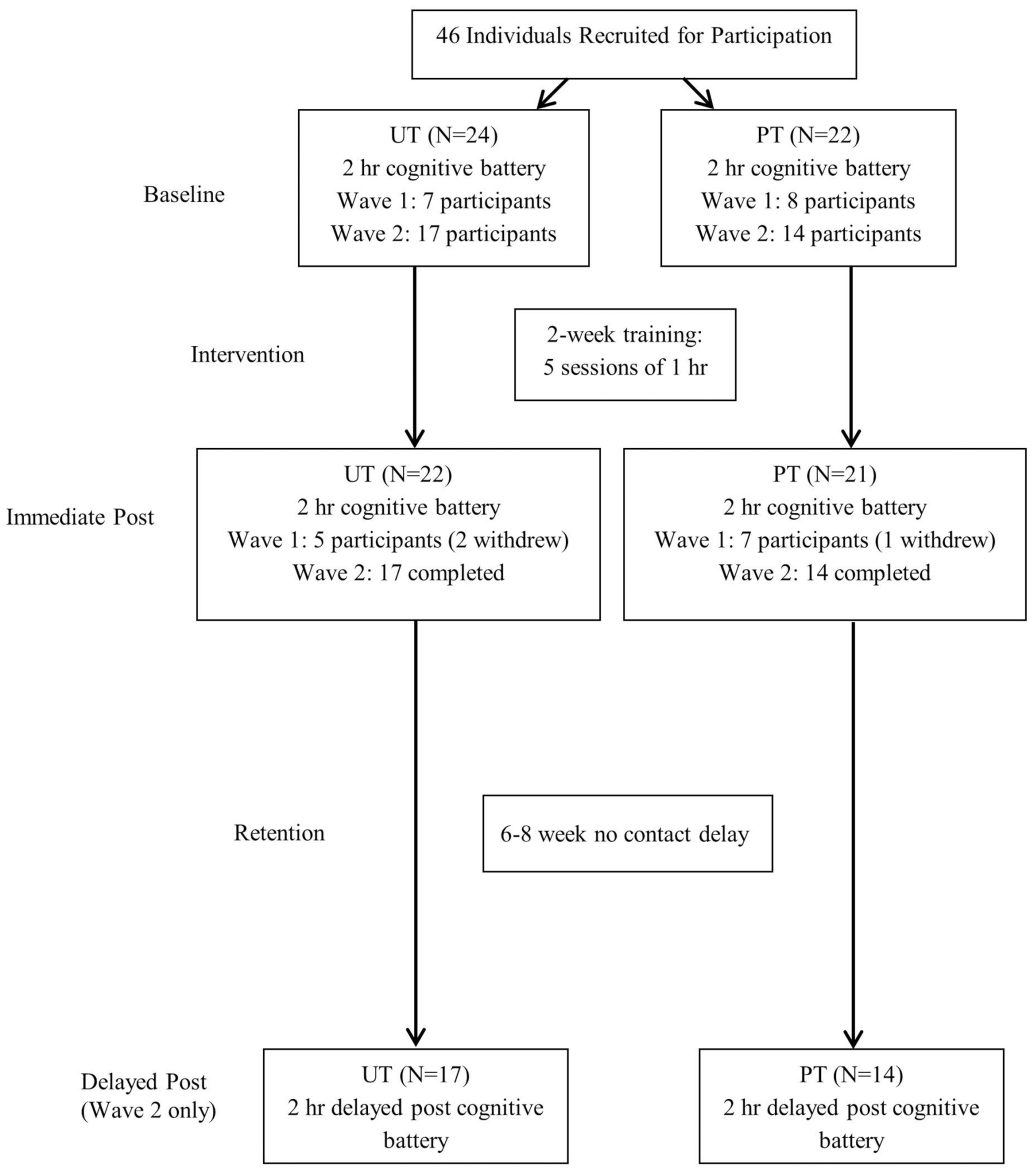
**Flow of participants through the training study.** UT, unpredictable training; PT, predictable training.

**Figure 2** shows the flow of participants and timeline of the study. This study was conducted in two waves, Wave 1 and Wave 2. Participants in Wave 2 were recruited over a longer period that included delayed post-testing. Forty-three participants were used in the analyses (*M*_age_ = 68.81, *SD*_age_ = 5.18, *M*_education_ = 15.05, *SD*_education_ = 2.54), because three participants withdrew from the experiment due to personal reasons (e.g., health).

### Apparatus

All computer-based cognitive tests were programmed in E-prime (Psychology Software Tools, Pittsburgh, PA, USA). The computer-based cognitive tests were collected on networked PC computers with 22” Dell P2213 monitors, set to a 60 Hz 1920 × 1080 resolution.

### Procedure

Participants were randomly assigned to one of the two types of training – PT or UT. Participants in the two training groups did not differ in age, education, gender, or mental status (see **Table 1**). The paradigms used for the two types of training were exactly the same with the exception of the temporal dynamics of the probe sequence, which were predictable for PT and unpredictable for UT. The differences in the temporal dynamics of probe retrieval between the two types of training engendered different levels of cognitive control – high control in UT and low control in PT. Participants were trained on the respective paradigms over five 1 h training days spanning across 2 weeks, such that 1 week had two sessions and the other week had three sessions. Prior to training, all participants completed a 2 h battery of transfer tasks that assessed their *baseline* pre-training performance. They underwent assessments of these transfer tasks again after the completion of their 2-week long training (*immediate post-testing*), allowing us to assess differential immediate post-test training benefits between the two groups. In addition to these two assessment sessions, in Wave 2, participants were asked to return back for a final assessment of the transfer tasks after a 1.5 month retention period (*delayed post-testing*). This allowed us to assess differential long-term benefits of training between the two groups. A small attrition rate of 6.52% was observed in this longitudinal 2-month study. No participant was provided any additional training during the retention period in Wave 2. Both groups of participants were told that they were participating in a training study. Therefore, there was no difference in motivation provided to the participants.

**Table 1:** Demographic information including mean (SD) of age, education and mental status.

Variable	Total	UT	PT
Sample size (Immediate; Delayed)	43; 31	22; 17	21; 14
Percentage of female	67.4	63.6	71.43
Age in years	68.81 (5.18)	68.82 (6.00)	68.81 (4.32)
Years of education	15.05 (2.54)	14.55 (2.77)	15.57 (2.23)
MMSE-2	28.84 (1.50)	28.68 (1.67)	29 (1.30)


*Mean differences for UT and PT groups were tested using independent samples *t*-test for continuous variables and chi-square tests for categorical variables. None of the tests were significant at *p* < 0.05.*

### Training Tasks: The N-Match Paradigm

The N-Match paradigm was adapted from our previously designed modified N-back (Verhaeghen et al., 2004; Verhaeghen and Basak, 2005) or random N-back (Basak and Verhaeghen, 2011a) paradigms. In these prior studies, the N differently colored digits were presented in N virtual columns, allowing for both color and location to act as retrieval cues. Multiple cues (location and color) in our modified N-back task facilitated retrieval latency and performance accuracy, when compared to a typical N-back task, where the items were presented in the same color and location (Vaughan et al., 2008). But the location cues require saccadic eye movements that can increase as a function of memory set-size, i.e., N. Therefore, in the current paradigm, all digits appeared in the same location at the center of the screen, with only color as a retrieval cue. That is, the current digit had to be compared with the digit presented immediately before in the same color. This allowed us to compare response times (RTs) of the predictable and the unpredictable versions of the N-Match task without saccadic latencies confounding the RTs, which in turn could exaggerate any differences between the two types of trained tasks. The N in the N-Match task represented the number of different colored information units that a participant had to simultaneously maintain and update during a trial run.

**FIGURE 3:**
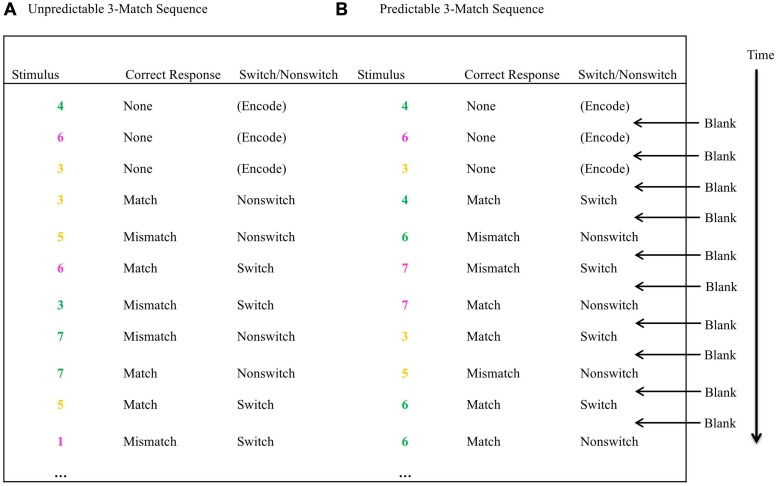
**Illustration of a three-Match trial run: (A) Unpredictable, where digits appeared in a random color sequence, (B) Predictable, where the digits appeared in the same color for two consecutive trials.** The first three trials were “encoding only” trials where no response was required, because there were no prior trials to compare with. The subsequent trials required responses. “Blank” represents a black screen presented for 300 ms to allow for distinction between two consecutively presented same-colored digits of equal identities.

Before each trial run of 40 trials, distinct encoding digits were shown sequentially in N different colors. N varied from 1 to 4, with the probe-color for *N* = 1 as yellow, *N* = 2 as yellow and pink, *N* = 3 as yellow, pink, and red, and *N* = 4 as yellow, pink, red, and green. After the N encoding digits were presented, probe digits were presented on the screen one at a time in one of the N colors. Participants had to compare the identity of the current digit with the digit shown *immediately* before in the same color. If the current digit matched the previously presented same-colored digit, the participant had to press the ‘z’ key with their left forefinger. If the two digits did not match, the ‘m’ key needed to be pressed with their right forefinger, and the previous digit in this color needed to be updated with the new one for subsequent comparison. Half of the trials required such updating. The task was self-paced, with a blank screen of 300 ms appearing before the onset of the next digit. This blank mask caused jittering, allowing the participants to distinctly perceive two subsequent identical digits of the same color. A digit stayed on the screen until a key press response was made (see **Figure 3A** for an illustration of a trial run). In line with previous research, the first N encoding-only trials were discarded and only the probe-recognition trials were retained for the analyses (Verhaeghen et al., 2004; Verhaeghen and Basak, 2005; Basak and Verhaeghen, 2011a). The digits were shown in size 18, Courier New font against a black background. Computers were placed approximately 80 cm from the participants.

For *N* = 1, the digits were presented in just one color. Therefore, the participants had to compare the current digit with the previously shown digit, making this equivalent to a typical 1-back task.

#### Unpredictable N-Match Paradigm

For *N* > 1, more than one colored digit was presented, necessitating a switch from one color cue to another. Multiple colored units also varied the temporal dynamics of probe presentation as a function of the type of training. The probe (i.e., the color) sequence in the unpredictable N-Match task was random. For example, for *N* = 3, after the initial encoding stimuli in three colors, viz., red (R), yellow (Y), and pink (P), were shown, a probe-color sequence could be RYYYPPYRRR (see **Figure 3A** for an example of an UT trial). Half of the trials in a trial run were switch trials, where the probe-color for the current digit was different from that of the previously presented digit in the sequence. As mentioned before, half of the trials in a trial run were also update trials.

#### Predictable N-Match Paradigm

The main difference between the unpredictable and predictable versions of the task was that in the latter the probe sequences were predictable. Again, half of the trials in a trial run were switch trials. This was achieved in each trial run by presenting each probe-color twice in a sequence before switching to another probe-color. For example, for *N* = 3, a probe-color sequence could be RRYYPPRRYY (see **Figure 3B** for an example of a PT trial). Such a sequence allowed for alternating switch and non-switch trials, unlike the predictable N-back paradigms used in prior research, which have always necessitated a switch from one color to another, and, therefore, had no non-switch trials (Verhaeghen et al., 2004; Verhaeghen and Basak, 2005). In sum, the only difference between the two types of training, UT vs. PT, was the probe-cue sequencing. The probability of switch, the proportion of trials requiring updating, the set-size (N), the type of retrieval-cue (color), number of blocks, and the amount of training (5 h) was equivalent for the two groups.

Irrespective of the type of training, each 1 h training day was divided into five blocks. In each block, the set-size (i.e., N) varied from small-to-large-to-small (i.e., 1-Match, 2-Match, 3-Match, 4-Match, 4-Match, 3-Match, 2-Match, 1-Match). This yielded 80 trails per N. In each training day, the first four blocks were on the training task, but the fifth block was on the predictable N-Match task (i.e., the *comparison task*). The motivation behind this block was to assess any difference in learning across the 5 days between the two groups in the lower cognitive control version of the task. This block of the *comparison task* was analyzed separately from the other four training task blocks.

### Transfer Tasks

Transfer tasks were selected using a construct approach; see **Table 2** for details about the tasks, constructs and forms used. The paper pencil tasks had two parallel forms to allow for multiple assessments.

**Table 2:** Transfer tasks completed by both training groups at baseline, immediately post training, and delayed post testing.

Task name	Session assessed	Construct measured	Transfer	Primary measure
DSST	1(A), 2(B), and 3(A)	Processing speed	–	# of pairs created in 30 s
SingleRT^∗^	1, 2, and 3	Processing speed	–	Choice reaction time
ForwardSpan	1, 2, and 3	Short-term memory	–	# correctly repeated in order
BackwardSpan	1, 2, and 3	Working memory/CC	Near	# correctly repeated in reverse order
DualSwitchCost^∗^	1, 2, and 3	CC	Near	Dual local switch cost
UnpredSwitchCost^∗^	1, 2, and 3	CC	Near	Unpredictable local switch cost
RAPM	1(A), 2(B), and 3(A)	Reasoning	Far	# correct
StoryRecall	1(A), 2(B), and 3(A)	Episodic Memory	Far	# of correctly recalled details


A and B refer to parallel forms of the task. *Computerized Tasks. CC = Cognitive Control.

#### Near Transfer Tasks

##### Backward span

Both forward and backward digit span tasks were taken from the Working Memory Index (WMI) in the Wechsler Adult Intelligence Scale (WAIS; Wechsler, 1939). Digits were presented verbally in an incremental set-size. Participants had to repeat the digits in the same order (forward) or the reverse order (backward) of the presentation. Backward span, in addition to encoding, storage and retrieval involved in forward span, requires coordination of information units. Therefore, it is considered to be a measure of working memory.

##### Task switching

The task switching paradigm utilized in this study is similar to that used in cognitive training (e.g., Kramer et al., 1999a; Basak et al., 2008), where the background color of the stimuli determined the task at hand. If the background was blue, participants indicated whether the digit presented was higher (‘z’ key) or lower (‘/’ key) than the digit 5. If the background was pink, participants indicated whether the digit was odd (‘z’ key) or even (‘/’ key). The digit 5 was never used and participants were required to use two hands to respond. The stimuli were presented in the center of the screen for 1500 ms. Participants completed two single task blocks, one for each task. This was followed by two multi-tasking blocks – one where the two tasks were interleaved predictably (e.g., Blue Blue Pink Pink Blue Blue…) and another where the tasks were interleaved randomly. The primary measure was the switch cost, i.e., the residuals obtained from regressing the average RT of non-switch trials from the average RT of switch trials. The *DualSwitchCost* was obtained from data of all trials, and the *UnpredSwitchCost* was calculated from data of just the unpredictable trials. These two switch costs were considered to be measures of cognitive control.

#### Far Transfer Tasks

##### Raven’s Advanced Progressive Matrices

In Raven’s Advanced Progressive Matrices (RAPM), participants were instructed to find the missing abstract pattern from a 3 × 3 matrix of complex visual designs. The missing pattern was one of eight possible choices that the participant was presented with. The full version of 36 items was divided into two sub-sets of 18 questions of the same difficulty level. Version A included the even questions from the first half and the odd questions from second half. Version B included the opposite combination of task questions. Participants were given 30 min to complete as many of the 18 abstract puzzles as possible. RAPM is an abstract reasoning task (Raven, 1942).

##### Story Recall

A short story from the MMSE-2: Expanded Version (with two parallel versions) was read out to the participants, who were prompted to remember as many details from the story. The number of correctly recalled details (with a maximum possible score of 25) was used as a measure of episodic memory (Folstein et al., 1975; Folstein et al., 2010).

Additional tasks, viz., Forward Span, Digit Symbol Substitution Test (DSST; from the MMSE-2: Expanded Version) and single RTs (from the single task blocks of the task switching paradigm), were assessed to establish whether training-related changes were due to improvements in processing speed or short-term memory capacity.

### Analysis Techniques to Assess Learning-related Changes in the Temporal Dynamics in the Trained Tasks

Individual learning rates for items within the FoA (i.e., 1-Match trials) and for items outside of the FoA (i.e., 2-, 3-, and 4-Match trials) were calculated by fitting power functions (*Y* = aX^b^) to the RTs across the five training days. Parameter b represented the rate of learning. Individual learning rates were assessed for the first four blocks of each training day to evaluate the differences in learning both inside and outside the FoA for the two types of training. This resulted in four learning rates, viz., inside the FoA for *N* = 1 trials for PT (*inFoA PT lng*), inside the FoA for *N* = 1 trials for UT (*inFoA UT lng*), outside the FoA for PT (*outFoA PT lng*), and outside the FoA for UT (*outFoA UT lng*). Moreover, data from the fifth block of each training day yielded learning rates for all participants on the comparison task, i.e., the predictable N-Match task.

## Results

Outlier corrections on a participant-by-participant basis for each condition were conducted by deleting trials with RT either below or above 3 *SD*; trials with RTs < 200 ms were also removed. Average RT for each individual, for each condition, was derived from accurate trials only. The alpha level for statistical significance was 0.05; *p*-values were Greenhouse-Geisser corrected for sphericity.

### Temporal Dynamics of Predictable vs. Unpredictable N-Match Task at Baseline

**FIGURE 4:**
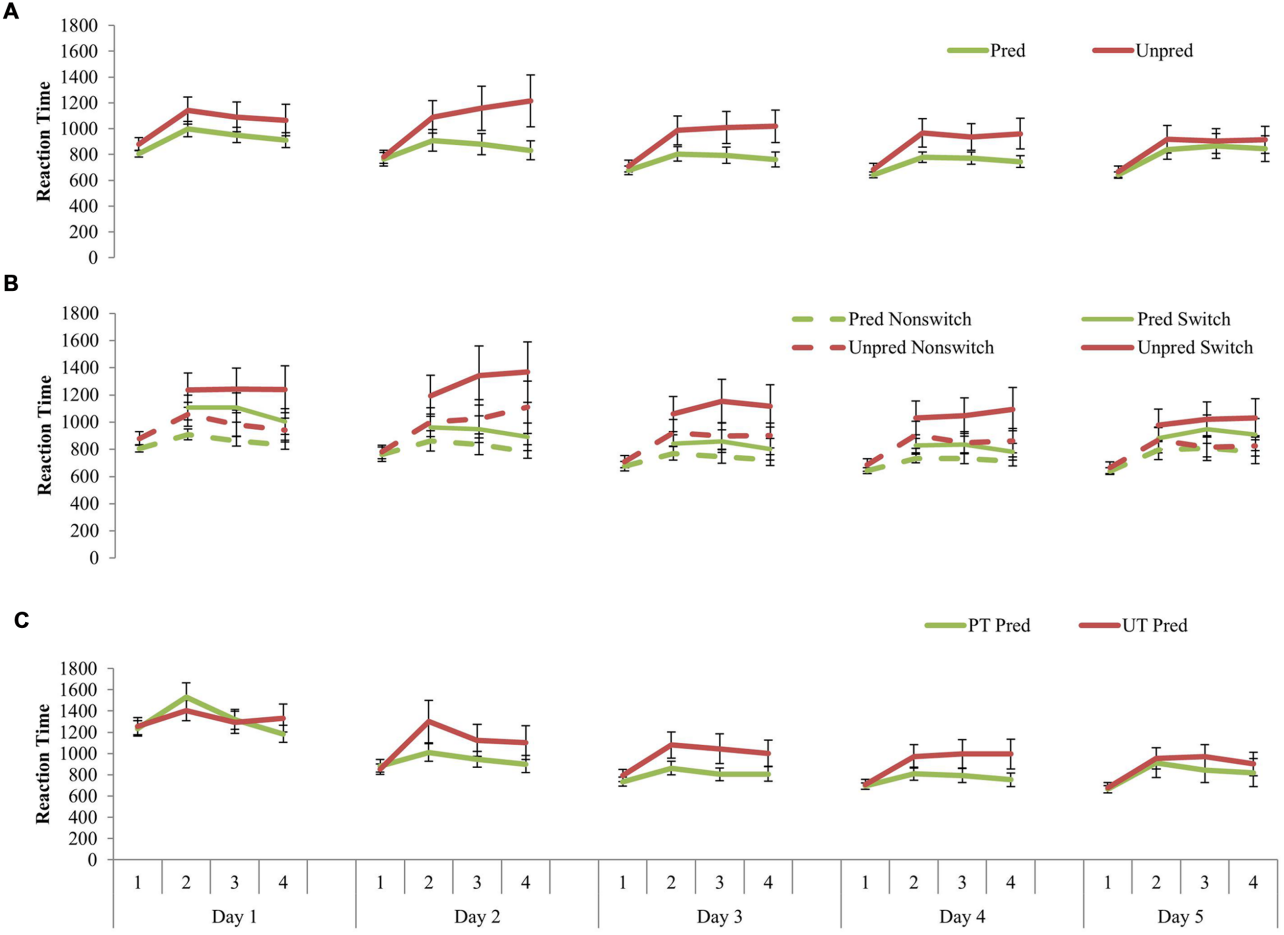
**(A)** Training-related changes in probe retrieval across 5 days of training. The unpredictable group became more similar to the predictable group on the final day of training (Day 5). **(B)** Training-related changes in probe retrieval for switch and non-switch trials across 5 days of training. Both training groups exhibited worse performance on the switch trials compared to the non-switch trials, even after 5 h of training. **(C)** Training-related changes in the comparison task after 5 days of training. The two groups did not differ. Error bars represent standard error of the mean. Pred, predictable; Unpred, unpredictable; PT, predictable training group; UT, unpredictable training group.

The temporal dynamics of the unpredictable and predictable tasks were investigated using Day 1’s RT performance in the four different Ns. First, a separate univariate repeated measures ANOVA, with set-size (*N* = 1, 2, 3, and 4) as a factor, was conducted for each training group. The main effect of N for the predictable group was significant, *F*(1.20,23.95) = 5.48, *p* = 0.02, *MSE* = 135840.88, $\eta_{p}^{2}$ = 0.22. *Post hoc* multiple comparisons, using the repeated contrasts, indicated that RTs of *N* = 2 were significantly slower than RTs of *N* = 1 by approximately 250 ms, *F*(1,20) = 8.82, *p* < 0.01, *MSE* = 159084.84, $\eta_{p}^{2}$ = 0.31. In the unpredictable task, the main effect of N was also significant, *F*(1.52,31.87) = 5.00, *p* = 0.02, *MSE* = 55489.48, $\eta_{p}^{2}$ = 0.19. RTs of *N* = 2 were significantly slower than RTs of *N* = 1 by approximately 200 ms, *F*(1,21) = 10.50, *p* = 0.004, *MSE* = 77863.52, $\eta_{p}^{2}$ = 0.33 (see **Figure 4A**: Day 1). Therefore, in both versions of the task, we found evidence of focus switch costs, indicated by a significant increase in RTs from *N* = 1 to 2. They are comparable to the focus switch costs from past studies on older adults in both the N-back and the random N-back tasks (240–500 ms; Verhaeghen and Basak, 2005; Vaughan et al., 2008; Basak and Verhaeghen, 2011a).

To test whether focus switching affected the temporal dynamics of items in the outer store, two separate 3 (*N* = 2, 3, and 4) × 2 (Switch_type: switch vs. non-switch) ANOVAs, one for UT and another for PT, were conducted on the Day 1 RTs. For the predictable task, the main effect of N was not significant, *F*(1.42,28.34) = 1.26, *p* = 0.28, *MSE* = 30260.24, $\eta_{p}^{2}$ = 0.06, suggesting that the focus switch cost remained unchanged outside the FoA. But the main effect of Switch_type was significant, *F*(1,20) = 10.26, *p* = 0.004, *MSE* = 198324.4, $\eta_{p}^{2}$ = 0.34, suggesting that the switch RTs were slower than the nons-witch RTs. Importantly, the N × Switch_type interaction was not significant, *F*(1.30,25.93) = 2.17, *p* = 0.15, *MSE* = 10040.62, $\eta_{p}^{2}$ = 0.10, suggesting no difference in RT slopes between the switch and non-switch trials. For the unpredictable task, both main effects of N, *F*(1.21,25.30) = 4.61, *p* = 0.04, *MSE* = 38685.07, $\eta_{p}^{2}$ = 0.18, and Switch_type, *F*(1,21) = 10.52, *p* = 0.004, *MSE* = 123867.72, $\eta_{p}^{2}$ = 0.33, were significant. Switch trials were slower than non-switch trials. *Post hoc* comparisons using repeated contrasts indicated that the RTs for *N* = 2 and *N* = 3 were the same (*F <*1), but were significantly faster for *N* = 4 compared to *N* = 3, *F*(1,21) = 8.99, *p <*0.01, *MSE* = 9334.36, $\eta_{p}^{2}$ = 0.30; the latter unexpected result could be due to speed-accuracy tradeoff evidenced by near-chance performance at *N* = 4 (60% accuracy). Importantly, the interaction was not significant, *F*(1.34,28.16) = 1.10, *p* = 0.32, *MSE* = 15778.77, $\eta_{p}^{2}$ = 0.05, indicating that the RT slopes of the switch and non-switch trials were the same.

### Learning-Related Changes in the Temporal Dynamics in the Trained Tasks

Differential learning-related changes in the temporal dynamics (RTs) of the probe retrieval across the 5 days were investigated using a 2 (Training_type) × 5 (Day) × 4 (N) ANOVA, where Day and N are within-subjects factors and Training_type was a between-subjects factor. The main effect of Day was significant, *F*(2.53,101.04) = 5.81, *p* = 0.002, *MSE* = 272139.62, $\eta_{p}^{2}$ = 0.13, suggesting that significant learning on the trained task happened over 5 days. *Post hoc* comparisons using repeated contrasts revealed significant differences between Days 2 and 3, *F*(1,40) = 4.50, *p* = 0.04, *MSE* = 101953.72, $\eta_{p}^{2}$ = 0.10, and a marginally significant difference between Days 3 and 4, *F*(1,40) = 3.59, *p* = 0.07, *MSE* = 15668.44, $\eta_{p}^{2}$ = 0.08, suggesting rapid learning after Day 2. The main effect of N was significant, *F*(1.24,49.73) = 15.28, *p* < 0.001, *MSE* = 370949.00, $\eta_{p}^{2}$ = 0.28. *Post hoc* comparisons using repeated contrasts indicated a significant focus switch cost from *N* = 1 to 2 regardless of the type or day of training, *F*(1,40) = 22.93, *p* < 0.001, *MSE* = 87390.72, $\eta_{p}^{2}$ = 0.36. The main effect of Training_type and its interactions with other variables were non-significant (*p*’s > 0.34). This suggests that the learning rates for the two versions of the N-Match task were equivalent over 5 days of training. This was corroborated by non-significant differences between the learning rates of the two types of training for both inside the FoA [-0.14 for PT vs. -0.18 for UT, *t*(41) = 0.85, *p* = 0.22] and outside the FoA [-0.12 for PT vs. -0.11 for UT, *t*(41) = 0.47, *p* = 0.65] (**Supplementary Figure S1**)^1^.

To test whether the switch and non-switch RTs changed differentially with extensive practice, a 2 (Training_type) × 5 (Day) × 3 (N = 2, 3, and 4) × 2 (Switch_type) ANOVA was conducted (see **Figure 4B**). The main effect of Day, *F*(2.58,103.35) = 3.90, *p* = 0.02, *MSE* = 661181.2, $\eta_{p}^{2}$ = 0.09, main effect of Switch_type, *F*(1,40) = 22.19, *p* < 0.001, *MSE* = 400251, $\eta_{p}^{2}$ = 0.36, Day × Switch_type interaction, *F*(2.04,81.67) = 3.96, *p* = 0.02, *MSE* = 41204.88, $\eta_{p}^{2}$ = 0.09, and N × Switch_type interaction, *F*(1.35,54.00) = 5.57, *p* = 0.01, *MSE* = 33036.89, $\eta_{p}^{2}$ = 0.12, were found to be significant. All other main effects and interactions were not significant. These results suggest that although the focus switch cost was greater for larger set-sizes, extensive practice brought greater improvements to the switch latencies than the non-switch latencies.

To assess whether the two training groups differed in the comparison (predictable) task, we conducted a 2 (Training_type) × 5 (Day) × 4 (*N* = 1, 2, 3, and 4) ANOVA. No main effect of Training_Type or its interactions with the other variables were significant, suggesting that UT was not worse than PT in the predictable N-Match task (**Figure 4C**).

To evaluate whether the FoA expanded with 5 h of practice, defined by negligible difference in switch and non-switch RTs at *N* ≥ 2, we conducted separate 3(*N* = 2, 3, and 4) × 2(Switch_type) ANOVAs for predictable and unpredictable RTs from the final day of the training (Day 5). The results were similar to those from Day 1. For both PT and UT groups, the main effect of N and the N x Switch_type interaction were not significant. But the main effect of Switch_type was significant; PT: *F*(1,19) = 6.97, *p* = 0.02, *MSE* = 73033.68, $\eta_{p}^{2}$ = 0.27; UT: *F*(1,21) = 16.18, *p* = 0.001, *MSE* = 50003.70, $\eta_{p}^{2}$ = 0.44. That is, even after 5 h of practice, the focus switch cost was significant in both versions of the task, suggesting that FoA still held only one information unit. This was corroborated by a significant difference in RTs between *N* = 1 and 2 at Day 5; PT: *t*(19) = -2.86, *p* = 0.01; UT: *t*(21) = -3.14, *p* < 0.01.

### Transfer of Training

Although learning-related changes in the two types of training did not vary, it is plausible that the high cognitive control training (UT) may engender greater transfer to unrelated untrained constructs, than low cognitive control training (PT). Our effect sizes, similar to previous training studies, were expected range from medium ($\eta_{p}^{2}$: 0.06 to 0.14) to large ($\eta_{p}^{2}$ > 0.14; Cohen, 1988; Tabachnick and Fidell, 2007). Means and standard deviations of the measures of the transfer tasks for PT and UT are provided in **Supplementary Table S1**. No significant difference was observed between UT and PT in any of the transfer measures at baseline (see Supplementary Materials).

**FIGURE 5:**
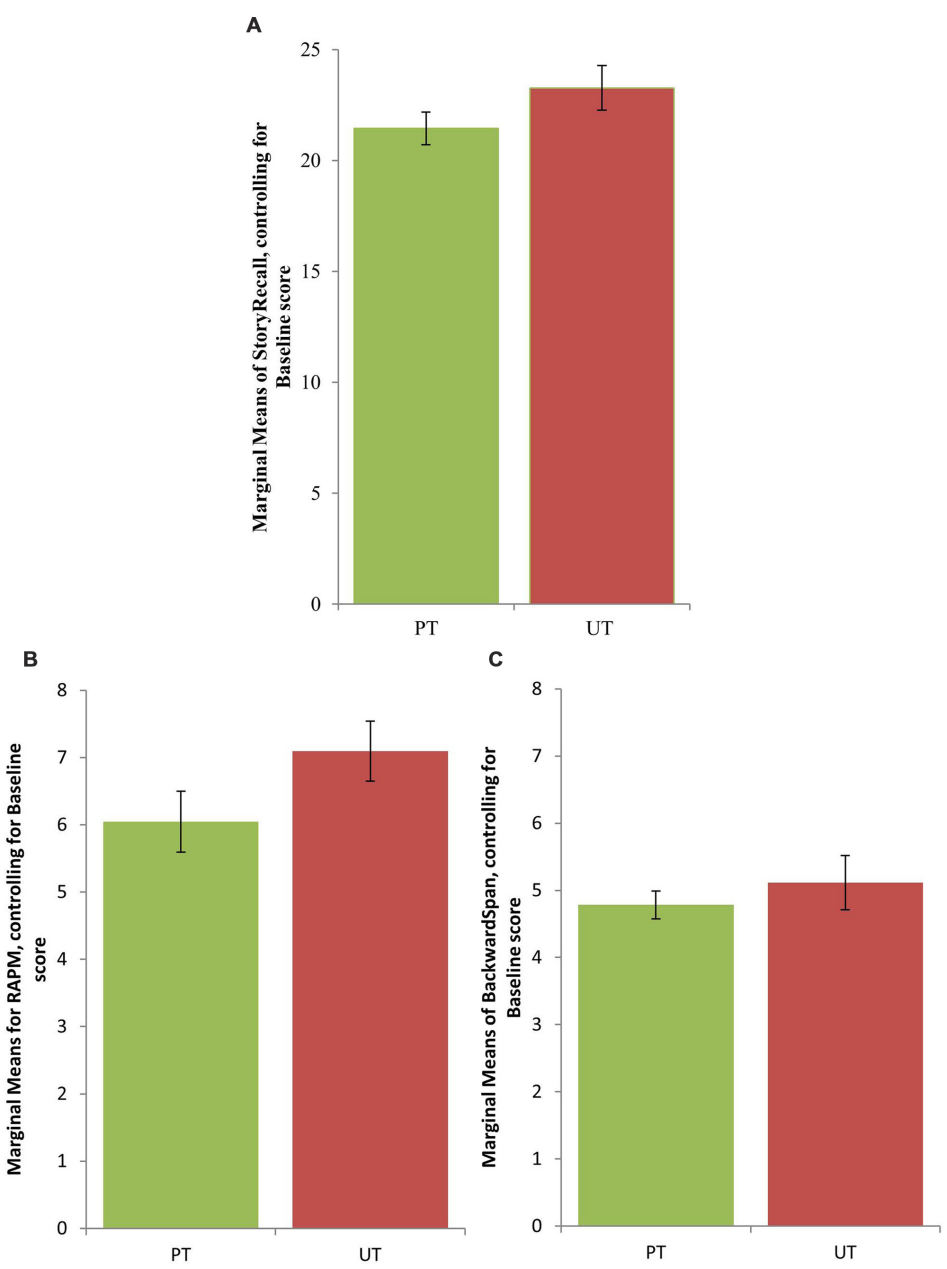
**Marginal means for **(A)** Story Recall, **(B)** Raven’s Advanced Progressive Matrices, and **(C)** Backward Span at immediate post-test session, controlling for the baseline performances.** Error bars represent standard error of the mean.

In line with previous intervention studies, separate analyses of covariance (ANCOVAs) were conducted on each transfer task measure to evaluate differences between the two training groups (PT vs. UT) at both immediate post testing as well as delayed post testing (e.g., Boot et al., 2010). In repeated measures analyses, any significant Training_type × Session interaction may be due to regression toward the mean, and not due to the differential training effects. Therefore, to ensure that any post-testing performance gains in the UT, compared to the PT, was not due to regression toward the mean, we used ANCOVA that accounted for individual differences at baseline. In addition, inFoA lng was used as a covariate, because it accounted for individual differences in changes in learning speed in the easiest condition (*N* = 1). This condition was common across the two training groups. Results are depicted in **Supplementary Table S2**. A medium effect, albeit of marginal significance, in favor of the UT group immediately after the training was found for two far transfer tasks: story recall, *F*(1,39) = 3.09, *p* = 0.09, *MSE* = 11.04, $\eta_{p}^{2}$ = 0.07, and RAPM, *F*(1,39) = 2.73, *p* = 0.10, *MSE* = 4.27, $\eta_{p}^{2}$ = 0.07 (**Figure 5**).

Another analysis technique to assess transfer effects is to pool scores from different testing sessions and then conduct a rank-ordered Blom transformation (Blom, 1958). Rank-order transformations, e.g., Tukey and Blom transformations, have previously been used to correct for within-variable errors (Knoke, 1991). Blom transformations, however, produce a better fit to the normal distribution than Tukey transformations (Bonate, 2000). Pooling of baseline and immediate post-test dependent measures and pooling of the dependent measures from all three testing sessions were conducted separately to take into account the varied number of participants in the immediate post-testing and the delayed post-testing sessions. This resulted in a slight difference in the ranking within the dependent variables. Blom transformed data allowed us to conduct repeated measures ANOVAs for each transfer task measure on normally distributed data. Separate analyses were conducted to compare immediate post vs. baseline and delayed post vs. baseline, with inFoA lng as a covariate. Thus, multiple 2 (Session) × 2 (Training_type) repeated measures ANOVAs were conducted on these data to assess the interaction effects. These analytic methods followed those conducted in previous cognitive training studies involving older adults, both for short-term targeted interventions lasting for 10 h (e.g., ACTIVE Study; Ball et al., 2002) or long-term non-targeted interventions spanning over months that were directed to change the lifestyle of older adults (Chan et al., 2014).

The results of the main effects and interactions are provided in **Table 3.** A medium effect size was observed for the Session × Training_type interaction at immediate post-test session favoring UT for the story recall task, *F*(1,40) = 4.23, *p* < 0.05, *MSE* = 0.39, $\eta_{p}^{2}$ = 0.10, and for the RAPM, *F*(1,40) = 2.38, *p* = 0.13, *MSE* = 0.23, $\eta_{p}^{2}$ = 0.06. No other tasks showed medium effects for the interaction term, either at immediate or delayed post-test sessions^2^.

**Table 3:** Repeated measures ANOVA calculated using Blom transformed measures at baseline vs. immediate-post training session, and baseline vs. delayed-post sessions.

	Immediate post	Delayed post
**DSST**		
M.E. Session	*F*(1,40) = 0.35, *p* = 0.56, *MSE* = 0.50, $\eta_{p}^{2}$ = 0.009	*F*(1,28) = 0.07, *p* = 0.78, *MSE* = 0.22, $\eta_{p}^{2}$ = 0.003
M.E. Condition	*F*(1,40) = 0.06, *p* = 0.81, *MSE* = 1.51, $\eta_{p}^{2}$ = 0.001	*F*(1,28) = 0.40, *p* = 0.53, *MSE* = 1.76, $\eta_{p}^{2}$ = 0.014
Session × Condition	*F*(1,40) = 0.05, *p* = 0.83, *MSE* = 0.50, $\eta_{p}^{2}$ = 0.001	*F*(1,28) = 0.00, *p* = 0.996, *MSE* = 0.22, $\eta_{p}^{2}$ = 0.000
**SingleRT**		
M.E. Session	*F*(1,39) = 0.37, *p* = 0.55, *MSE* = 0.58, $\eta_{p}^{2}$ = 0.009	*F*(1,27) = 0.15, *p* = 0.71, *MSE* = 0.68, $\eta_{p}^{2}$ = 0.005
M.E. Condition	*F*(1,39) = 0.40, *p* = 0.53, *MSE* = 1.44, $\eta_{p}^{2}$ = 0.010	*F*(1,27) = 0.53, *p* = 0.47, *MSE* = 1.17, $\eta_{p}^{2}$ = 0.019
Session × Condition	*F*(1,39) = 0.82, *p* = 0.37, *MSE* = 0.58, $\eta_{p}^{2}$ = 0.020	*F*(1,27) = 0.02 *p* = 0.90, *MSE* = 0.68, $\eta_{p}^{2}$ = 0.001
**ForwardSpan**		
M.E. Session	*F*(1,40) = 3.87, *p* = 0.06, *MSE* = 0.34, $\eta_{p}^{2}$ = 0.088	*F*(1,28) = 0.01, *p* = 0.91, *MSE* = 0.74, $\eta_{p}^{2}$ = 0.000
M.E. Condition	*F*(1,40) = 0.94, *p* = 0.34, *MSE* = 1.33, $\eta_{p}^{2}$ = 0.023	*F*(1,28) = 0.76, *p* = 0.39, *MSE* = 1.12, $\eta_{p}^{2}$ = 0.027
Session × Condition	*F*(1,40) = 0.41, *p* = 0.53, *MSE* = 0.34, $\eta_{p}^{2}$ = 0.010	*F*(1,28) = 0.11, *p* = 0.74, *MSE* = 0.74, $\eta_{p}^{2}$ = 0.004
**BackwardSpan**		
M.E. Session	*F*(1,40) = 0.20, *p* = 0.66, *MSE* = 0.45, $\eta_{p}^{2}$ = 0.005	*F*(1,28) = 1.04, *p* = 0.32, *MSE* = 0.30, $\eta_{p}^{2}$ = 0.036
M.E. Condition	*F*(1,40) = 0.48, *p* = 0.49, *MSE* = 1.29, $\eta_{p}^{2}$ = 0.012	*F*(1,28) = 0.57, *p* = 0.48, *MSE* = 1.63, $\eta_{p}^{2}$ = 0.018
Session × Condition	*F*(1,40) = 0.73, *p* = 0.40, *MSE* = 0.45, $\eta_{p}^{2}$ = 0.018	*F*(1,28) = 0.51, *p* = 0.48, *MSE* = 0.30, $\eta_{p}^{2}$ = 0.018
**DualSwitchCost**		
M.E. Session	*F*(1,39) = 0.16, *p* = 0.69, *MSE* = 0.32, $\eta_{p}^{2}$ = 0.004	*F*(1,27) = 0.47, *p* = 0.50, *MSE* = 0.36, $\eta_{p}^{2}$ = 0.017
M.E. Condition	*F*(1,39) = 0.35, *p* = 0.56, *MSE* = 1.72, $\eta_{p}^{2}$ = 0.009	*F*(1,27) = 1.86, *p* = 0.18, *MSE* = 1.50, $\eta_{p}^{2}$ = 0.064
Session × Condition	*F*(1,39) = 1.35, *p* = 0.25, *MSE* = 0.32, $\eta_{p}^{2}$ = 0.034	*F*(1,27) = 0.13, *p* = 0.72, *MSE* = 0.36, $\eta_{p}^{2}$ = 0.005
**UnpredSwitchCost**		
M.E. Session	*F*(1,39) = 0.80, *p* = 0.38, *MSE* = 0.38, $\eta_{p}^{2}$ = 0.020	*F*(1,27) = 0.03, *p* = 0.87, *MSE* = 0.38, $\eta_{p}^{2}$ = 0.001
M.E. Condition	*F*(1,39) = 0.20, *p* = 0.67, *MSE* = 1.65, $\eta_{p}^{2}$ = 0.005	*F*(1,27) = 0.42, *p* = 0.52, *MSE* = 1.45, $\eta_{p}^{2}$ = 0.015
Session × Condition	*F*(1,39) = 1.28, *p* = 0.27, *MSE* = 0.38, $\eta_{p}^{2}$ = 0.032	*F*(1,27) = 0.41, *p* = 0.53, *MSE* = 0.38, $\eta_{p}^{2}$ = 0.015
**RAPM**		
M.E. Session	*F*(1,40) = 0.64, *p* = 0.43, *MSE* = 0.23, $\eta_{p}^{2}$ = 0.016	*F*(1,28) = 1.04, *p* = 0.32, *MSE* = 0.30, $\eta_{p}^{2}$ = 0.036
M.E. Condition	*F*(1,40) = 0.14, *p* = 0.71, *MSE* = 1.75, $\eta_{p}^{2}$ = 0.004	*F*(1,28) = 0.52, *p* = 0.48, *MSE* = 1.63, $\eta_{p}^{2}$ = 0.018
Session × Condition	*F*(1,40) = 2.38, *p* = 0.13, *MSE* = 0.23, $\eta_{p}^{2}$ = 0.056	*F*(1,28) = 0.51, *p* = 0.48, *MSE* = 0.30, $\eta_{p}^{2}$ = 0.018
**StoryRecall**		
M.E. Session	*F*(1,40) = 0.27, *p* = 0.61, *MSE* = 0.39, $\eta_{p}^{2}$ = 0.007	*F*(1,28) = 10.19, *p* < 0.01, *MSE* = 0.51, $\eta_{p}^{2}$ = 0.267
M.E. Condition	*F*(1,40) = 0.08, *p* = 0.78, *MSE* = 1.15, $\eta_{p}^{2}$ = 0.002	*F*(1,28) = 0.48, *p* = 0.50, *MSE* = 0.75, $\eta_{p}^{2}$ = 0.017
Session × Condition	*F*(1,40) = 4.23, *p* = 0.046, *MSE* = 0.39, $\eta_{p}^{2}$ = 0.096	*F*(1,28) = 0.68, *p* = 0.42, *MSE* = 0.51, $\eta_{p}^{2}$ = 0.024


*Session refers to Testing_session.*

### Individual Differences in Gain Scores, Type of Training, and Learning Rates

**FIGURE 6:**
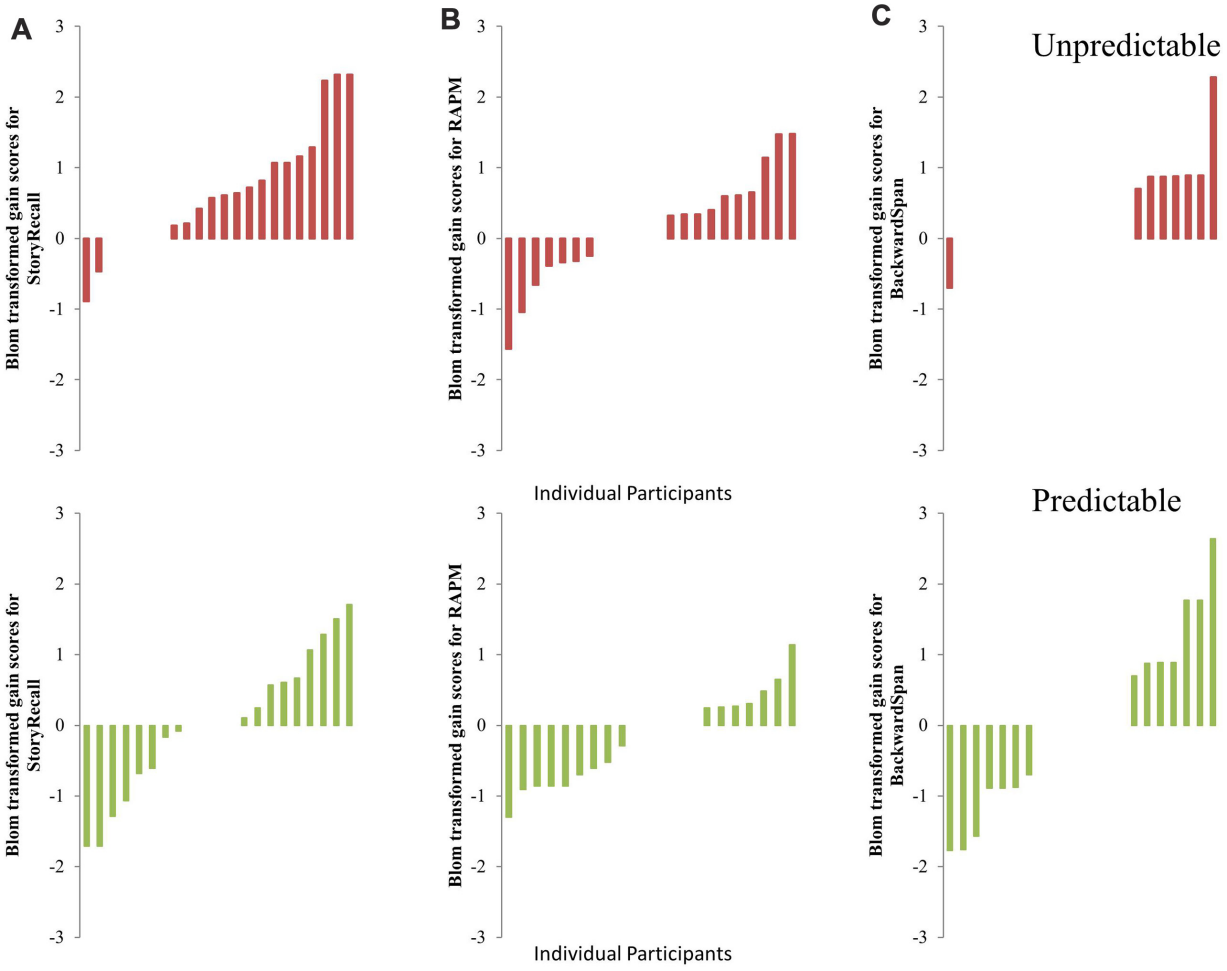
**Individual differences for Blom Transformed gain scores in **(A)** Story Recall, **(B)** RAPM, and **(C)** Backward Span for UT (Top) and PT (Bottom) groups.** Positive scores indicated performance gains and negative scores indicated performance declines after completion of training. Gains, favoring UT, were observed for Story Recall and RAPM.

In this secondary set of analyses, gain scores for each individual in all transfer tasks were calculated by subtracting their baseline Blom transformed score from their immediate post-test Blom transformed score. Positive scores indicated a training-related gain in the performance of the transfer task, whereas negative scores indicated a training-related loss. A larger number of UT participants had positive gain scores, compared to PT participants, in the two tasks that had yielded medium effect-sizes of transfer in the prior analyses, viz., Story Recall (**Figure 6C**) and RAPM (**Figure 6B**). In contrast, we found no observable differences in gain scores between UT and PT in backward span, a near-transfer task of working memory (**Figure 6C**). The differences in the number of participants who exhibited a positive gain score were greatest for the story recall.

**FIGURE 7:**
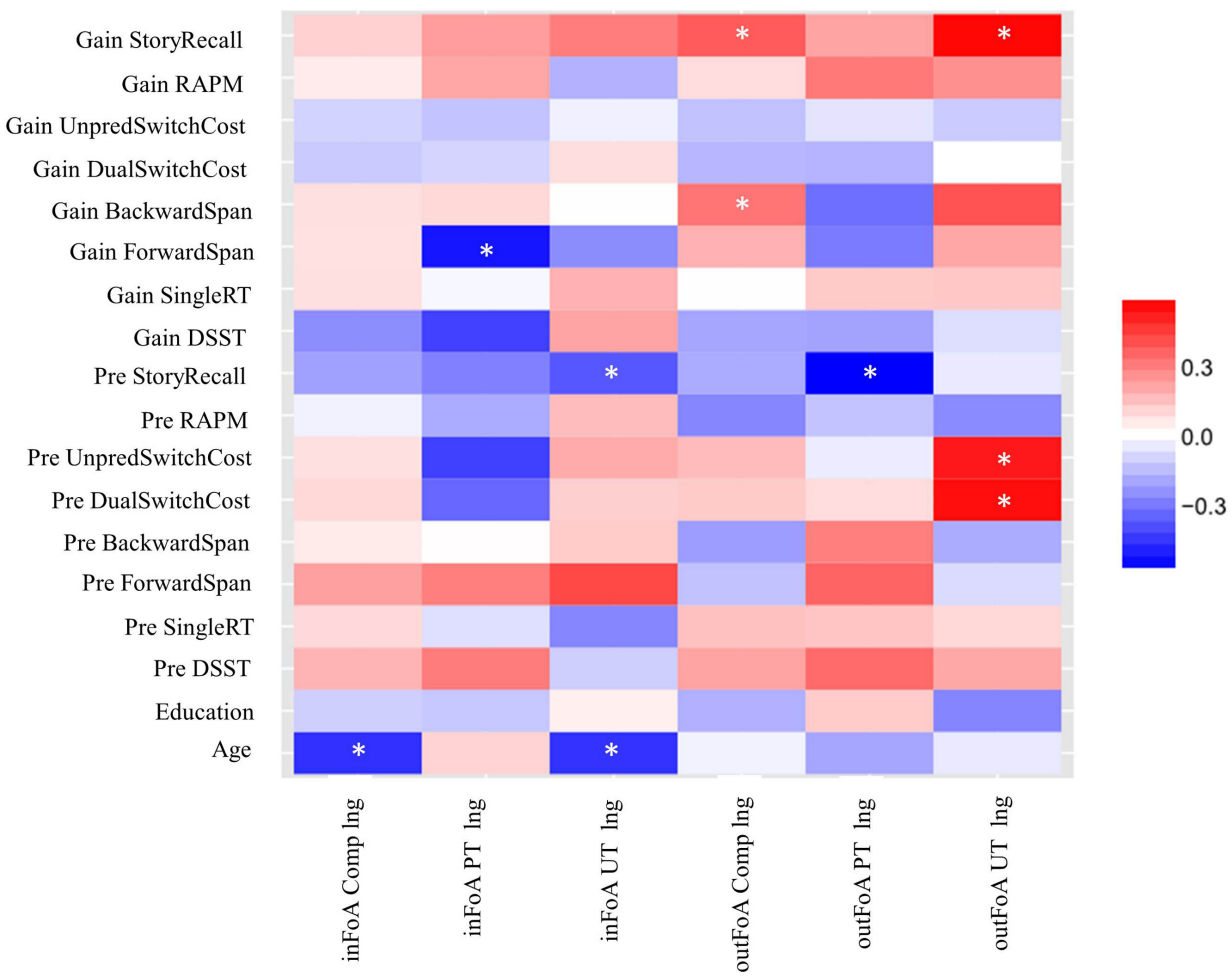
**A heat map representing correlation (*rho*) between the transfer tasks (both initial performances as well as training-based performance gains) and learning rates (the two training tasks for PT and UT separately and the comparison task for all participants).** An asterisk (^∗^) indicates a significant correlation.

It is plausible that the individuals who showed positive gains also started with higher cognitive abilities or had a greater general learning ability. On the other hand, if UT is a better strategy to induce transfer then the correlation between gain scores and learning rate may be significantly higher for UT than PT. Individual gain scores for the transfer tasks, baseline performances of the transfer tasks, previously described four learning rates, and the two learning rates for the comparison task (*inFoA Comp lng*, *outFoA Comp lng*) were subjected to non-parametric bivariate correlation analyses, represented by a heat map (**Figure 7**), where the strength of these relationships vary by the warmth of the color. Darker reds indicate a stronger positive correlation, whereas darker blues indicate a stronger negative correlation. All measures of RT were reverse coded (since slower RTs represent poorer performance). Therefore, in **Figure 7**, larger individual scores on each variable represented better performance.

In the UT group, significant positive correlations were observed between learning rates for the outer store and some of the measures of the transfer tasks, particularly, gain in story recall (*r* = 0.56, *p* = 0.01), baseline unpredictable switch cost (*r* = 0.52, *p* = 0.02), and baseline dual switch cost (*r* = 0.55, *p* = 0.01). Additionally, a marginally significant correlation was observed between learning rates for the outer store and gains in backward span (*r* = 0.39, *p* = 0.07). No significant positive relationships were observed for individuals in the PT group, even at *p* = 0.01. Therefore, individuals with greater changes in the temporal dynamics of the unpredictable task showed larger gains in story recall and, to some extent, backward span.

Using Fisher *r*-to-*z* transformation, we assessed whether the correlation coefficients for UT were higher than PT. For baseline scores in task-switching, the correlation coefficients were significantly higher for UT than PT: dual switch cost (*z* = 1.61, *p* = 0.05), unpredictable switch cost (*z* = 1.88, *p* = 0.03). For gain scores in the transfer tasks, the correlation coefficient was significantly higher for UT than PT in the backward span (*z* = 2.36, *p* < 0.01), and was marginally higher for UT than PT in the story recall (*z* = 1.28, *p* = 0.10).

When the two groups were combined in the comparison task, it provided us with more power to assess positive correlations between general learning rate in the N-Match task and the other cognitive tasks. Significant positive correlations were observed between learning rates for the outer store in the comparison task and performance gains in selected transfer tasks (story recall, *r* = 0.37, *p* = 0.01; backward span, *r* = 0.32, *p* = 0.04), although these results could be driven by the UT group.

## Discussion

We found selective transfer effects to the tasks of fluid cognition in the older adults who were trained on our novel memory updating approach, i.e., unpredictable probe-cue expectancies, compared to those older adults, who were trained on the standard predictable paradigm. Our conclusions on group differences on the transfer effects were based on the effect sizes, because our research questions were formulated in the estimation terms (Cumming, 2014). This is in line with recent research that has brought up issues of replication in psychology (Open Science Collaboration, 2015). Immediate post-testing transfer effects of medium effect size favoring the UT were found in *story recall*, a verbal episodic memory task, evidenced from two different types of planned analyses that have been typically used in prior studies of cognitive training, viz., repeated measures ANOVA on the Blom transformed data and analyses of covariance (ANCOVA), where baseline scores are accounted for. We also observed medium effect size of transfer effects, albeit not significant at *p* = 0.05, immediately after training favoring the UT training in *RAPM* in the ANCOVA analyses. Therefore, we consider these results to be weaker in comparison to those from the story recall. These differential selective transfer effects could not be merely explained by speeded learning of the trained task by the UT group, compared to the PT group. Because the two training tasks were similar in all aspects (e.g., switch probability, updating probability, set-size, stimulus-response relationship, identity judgment), but one (probe-cue expectancy), we expected to find smaller effect sizes of transfer than typical working memory training studies. Yet, for story recall, a medium effect size of immediate transfer was observed in both the ANCOVA and repeated measures analyses, favoring the UT group.

These results are in accordance with the Theory of Working Memory Adaptability (ToWMA) that proposes that cognitive control is the mechanism of transfer in working memory training. ToWMA predicts that the focus switch costs encountered when shifting between actively manipulated items are greater for sequences where probe-cue expectancies are more unpredictable. That is, unpredictable probe-cue sequences are hypothesized to require greater cognitive control than predictable probe-cue sequences, especially in older adults who have a marked deficit in cognitive control. If enhanced cognitive control in working memory is the mechanism of transfer in older adults, then unpredictable, when compared to predictable, training may show greater benefit to tasks of fluid cognition that are argued to be subserved by cognitive control. This hypothesis was supported by our ANCOVA/ANOVA analyses on the differential improvements in the transfer tasks, where UT had a more selective effect of transfer, which was medium-sized, to story recall than PT. Additionally, individual gain plots in **Figure 6** showed that more individuals in the novel, UT group had positive immediate performance gains in story recall (and RAPM) than the standard, PT group. These results indicate that just 5 h of training on unpredictable memory updating probe-cue expectancies may engender greater transfer to selective untrained skills than PT. Since the differential effect sizes of transfer favoring the novel training were at most medium, more studies are needed to further test the role of cognitive control in working memory training. They could explore the effects of training dose (e.g., 10 h instead of 5 h), consider multiple tasks of episodic memory, and/or compare our novel training paradigm to other types of working memory training approaches, where improved cognitive control could not be argued as the underlying mechanism of transfer.

In addition to conducting group-difference analyses to assess performance improvements at post training compared to the baseline by using ANOVAs/ANCOVAs, we also investigated the relationships between an individual’s ability to learn the trained tasks and gains in untrained cognition. It is plausible that individuals with higher cognitive abilities or learning ability may show the most cognitive gains irrespective to the type of working memory training. On the other hand, if UT is a better strategy to enhance cognition, then the correlation between the gain scores and the learning rates may be limited to the individuals who received the UT. Since accuracy for all individuals for the items inside the focus of attention (i.e., *N* = 1) was near perfect from Day 1, we were interested in the individual differences in the learning rates for items outside the focus of attention, where focus switching was necessitated between the actively held information units. In the novel UT group, greater efficiency in the temporal dynamics for manipulating and updating items in the unpredictable task was found to be significantly associated with larger gains in the story recall, a verbal episodic memory task, and marginally significantly associated with larger gains in the backward span, a near transfer working memory task. To counteract the issue of small sample size for these correlation analyses, Fisher’s *z*-tests were conducted to compare the correlation coefficients between the two training groups. The correlation coefficient in UT, compared to the PT, was significantly higher for backward span and marginally higher for story recall. Given that none of the measures of learning rates were significantly different between UT and PT (see **Supplementary Figure S1A**), the results from the *z*-tests supported ToWMA’s prediction that UT may be a better strategy to improve fluid cognition (e.g., backward span, story recall).

Also, according to ToWMA, unpredictable probe-cue expectancy training engenders greater demands on cognitive control than PT. In line with this theory, we found that the participants who exhibited more efficient cognitive control before the training, evidenced from the unpredictable switch costs and dual switch costs of task switching paradigm, learned the UT task faster. No such relationships were observed in the PT group. Furthermore, correlation coefficients between these baseline measures and learning rates of the trained tasks were significantly higher for UT than PT. Though these were exploratory analyses and the lack of correlation in PT group can be attributed to small sample size, we argue that such analyses should be incorporated in future studies. They would provide a better understanding of who would benefit most from a specific training strategy, and allow us to devise the best strategies to improve cognition in older adults. So far, our exploratory analyses suggest that older adults with more efficient cognitive control may show larger gains in selective tasks of fluid cognition with unpredictable memory updating training.

Although training working memory has gained popularity in the last decade, the mechanism of transfer that would allow us to determine the best strategies to improve cognition has not yet been systematically studied. In training studies, be it cognitive or fitness training, participants are typically recruited and assigned to either the training or a control group. Unlike fitness training studies where the controls are trained on a different type of fitness (e.g., aerobic compared to anaerobic; Erickson et al., 2012), cognitive training studies are fraught with issues regarding the use of an appropriate control group. The type of control group in any clinical trial allows us to make inferences about the power of the training-related benefits (Boot et al., 2011). If the control group consists of a no-contact control, then we cannot determine the mechanism of transfer. It is possible that mere participation in a training program induces cognitive benefits and, therefore, comparing working memory training with a passive control group does not provide us with knowledge on whether it is the training of working memory *per se* that is improving cognition. Yet, many recent studies have continued to use no-contact controls to compare against the working memory training group (Zinke et al., 2012, 2014; Heinzel et al., 2014; Stepankova et al., 2014). This methodological issue is not evident in other types of cognitive training, e.g., video game training (for a review, see Boot et al., 2011). In contrasts, if a study uses a placebo group as a control, e.g., social engagement or questionnaires (Borella et al., 2010, 2014; Carretti et al., 2013), it is plausible that cognitive benefits between the training group and the control group are associated with the participant’s belief that the experimental treatment should have an effect. Moreover, often in active control studies, experimental group is trained on an individualized adaptive paradigm, whereas the control group is trained on a non-adaptive paradigm (e.g., variable priority training vs. fixed-priority training). Choosing an appropriate control group will, therefore, have implications on the perceived benefits of the training. Although the results of transfer in younger adults have been mixed, plausibly driven by the type of control groups, working memory training compared to active controls do result in improvements on tasks of *near transfer* (see Melby-Lervåg and Hulme, 2013, for a meta-analytic review of study-specific effect sizes for different measures of transfer tasks). Importantly, a recent meta-analysis on age-related differences and cognitive training found that older adults trained in either working memory or task switching paradigms, both “core” abilities, benefitted in both near and far transfer tasks when compared to active controls (Karbach and Verhaeghen, 2014).

The results from the current study further our understanding of cognitive optimization in older adults by comparing two different types of working memory training – one novel approach that requires greater cognitive control (unpredictable probe-cue sequence) and another similar to previous training paradigms (e.g., N-back) that requires predictable focus switching. It is important to note that, in the current study, the two training paradigms were not individualized adaptive and were trained on the same updating task, varying in all but one dimension, viz. probe cue predictability. Therefore, any differences in our outcome variables favoring the novel training approach cannot be merely attributed to motivation or perceived benefits of training.

This study was not aimed to resolve the ongoing debate about whether, or not, working memory training in younger adults improves intelligence. We did not have an additional control group that would allow for assessment of the overall benefits of working memory training over a different type of training (e.g., Redick et al., 2013). Moreover, we consider our results indicating differential benefits to RAPM, a measure of non-verbal intelligence, to be weak at best. In the future, studies could compare multiple training groups that would allow for such assessments in addition to further investigation on the role of cognitive control in working memory training. These studies should also include larger number of participants and multiple measures of psychological constructs to allow for an investigation of individual differences in learning and transfer.

This study is important for the field of cognitive training because it furthers our understanding of the principles of cognitive optimization that is theory-driven, expounds the role of cognitive control in working memory, and helps us develop better cognitive training strategies in older adults. Moreover, the N-Match task was easier-to-learn and its training benefits were observed after a short-training period (5 h over 2 weeks), unlike other successful cognitive training studies used in older adults where either the training task was complex (e.g., video games; Basak et al., 2008), or intensive hours of training over a long period was required (e.g., 14 weeks at 15 h/week; Park et al., 2014). Although we failed to observe any long-term benefits of UT, compared to predictable, future studies could investigate how the length of training (5 vs. 10 h), amount of feedback, and frequency of training, influence long-term benefits of UT. Because improvements in episodic memory can delay the onset of Alzheimer’s disease, and our UT approach benefitted a task of episodic memory, future studies could use this novel training approach on at-risk individuals, such as, patients with Mild Cognitive Impairments or older adults with lower education, to investigate whether varying temporal dynamics of memory probe-cues during cognitive training can delay the onset of Alzheimer’s disease.

## Author Contributions

CB designed the research and programmed the experiments; CB and MO performed the research, analyzed the data and wrote the paper.

## Conflict of Interest Statement

The authors declare that the research was conducted in the absence of any commercial or financial relationships that could be construed as a potential conflict of interest.

## Footnotes

1 Also, the two learning rates in the comparison task, viz., *inFoA Comp lng* and *outFoA Comp lng*, did not differ between the two types of training.

2 A JZS Bayes factor Repeated Measure ANOVA (Rouder et al., 2012; Love et al., 2015; Morey and Rouder, 2015) was implemented to support these findings. For story recall, the interaction model between Session (baseline vs. immediate post) and Training_type was preferred to the main effects models by a Bayes factor of 1.452.

## References

[B1] Ball K. Berch D. B. Helmers K. F. Jobe J. B. Leveck M. D. Marsiske M. (2002). Effects of cognitive training interventions with older adults - a randomized controlled trial. *J. Am. Med. Assoc.* 288 2271–2281. 10.1001/jama.288.18.2271 PMC2916176 12425704

[B2] Basak C. (2006). Capacity limits of the focus of attention and dynamics of the focus switch cost in the working memory. [Dissertation Abstract]. *Diss. Abstr. Int. Sec. B Sci. Eng.* 66 5717.

[B3] Basak C. Boot W. R. Voss M. W. Kramer A. F. (2008). Can training in a real-time strategy video game attenuate cognitive decline in older adults? *Psychol. Aging* 23 765–777. 10.1037/a0013494 19140648 PMC4041116

[B4] Basak C. Verhaeghen P. (2011a). Three layers of working memory: focus-switch costs and retrieval dynamics as revealed by the N-count task. *J. Cogn. Psychol.* 23 204–219. 10.1080/20445911.2011.481621 PMC4375963 25821579

[B5] Basak C. Verhaeghen P. (2011b). Aging and switching the focus of attention in working memory: age differences in item availability but not in item accessibility. *J. Gerontol. Ser. B Psychol. Sci. Soc. Sci.* 66B, 519–526. 10.1093/geronb/gbr028 21571704 PMC3155026

[B6] Basak C. Voss M. W. Erickson K. I. Boot W. R. Kramer A. F. (2011). Regional differences in brain volume predict the acquisition of skill in a complex real-time strategy videogame. *Brain Cogn.* 76 407–414. 10.1016/j.bandc.2011.03.017 21546146 PMC4955609

[B7] Basak C. Zelinski E. (2013). “A hierarchical model of working memory and its change in healthy older adults,” in *Working Memory: The Connected Intelligence*, eds Alloway T. P. Alloway R. G. (London: Psychology Press), 83–106.

[B8] Bherer L. Kramer A. F. Peterson M. S. Colcombe S. Erickson K. Becic E. (2005). Training effects on dual-task performance: are there age-related differences in plasticity of attentional control? *Psychol. Aging* 20 695–709. 10.1037/0882-7974.20.4.695 16420143

[B9] Blom G. (1958). *Statistical Estimates and Transformed Beta-Variables.* New York, NY: John Wiley & Sons.

[B10] Bonate P. L. (2000). *Analysis of Pretest-Posttest Designs.* Boca Raton, FL: CRC Press.

[B11] Boot W. R. Basak C. Erickson K. I. Neider M. Simons D. J. Fabiani M. (2010). Transfer of skill engendered by complex task training under conditions of variable priority. *Acta Psychol.* 135 349–357. 10.1016/j.actpsy.2010.09.005 20920812

[B12] Boot W. R. Blakely D. P. Simons D. J. (2011). Do action video games improve perception and cognition? *Front. Psychol.* 2:226 10.3389/fpsyg.2011.00226 PMC3171788 21949513

[B13] Borella E. Carretti B. Cantarella A. Riboldi F. Zavagnin M. De Beni R. (2014). Benefits of training visuospatial working memory in young-old and old-old. *Dev. Psychol.* 50 714–727. 10.1037/a0034293 24059254

[B14] Borella E. Carretti B. Riboldi F. De Beni R. (2010). Working memory training in older adults evidence of transfer and maintenance effects. *Psychol. Aging* 25 767–778. 10.1037/a0020683 20973604

[B15] Carretti B. Borella E. Zavagnin M. de Beni R. (2013). Gains in language comprehension relating to working memory training in healthy older adults. *Int. J. Geriatr. Psychiatry* 28 539–546. 10.1002/gps.3859 22821686

[B16] Chan M. Y. Haber S. Drew L. M. Park D. C. (2014). Training older adults to use tablet computers: does it enhance cognitive function? *Gerontologist* 10.1093/geront/gnu057 [Epub ahead of print]. PMC4873760 24928557

[B17] Cohen J. (1988). *Statistical Power for the Social Sciences.* Hillsdale, NJ: Laurence Erlbaum and Associates.

[B18] Cowan N. (1988). Evolving conceptions of memory storage, selective attention, and their mutual constraints within the human information-processing system. *Psychol. Bull.* 104 163–191. 10.1037/0033-2909.104.2.163 3054993

[B19] Cowan N. (2001). The magical number 4 in short-term memory: a reconsideration of mental storage capacity. *Behav. Brain Sci.* 24 87–114. 10.1017/s0140525x01003922 11515286

[B20] Cumming G. (2014). The new statistics: why and how. *Psychol. Sci.* 25 7–29. 10.1177/0956797613504966 24220629

[B21] Dahlin E. Neely A. S. Larsson A. Backman L. Nyberg L. (2008). Transfer of learning after updating training mediated by the striatum. *Science* 320 1510–1512. 10.1126/science.1155466 18556560

[B22] Dubois B. Feldman H. H. Jacova C. DeKosky S. T. Barberger-Gateau P. Cummings J. (2007). Research criteria for the diagnosis of Alzheimer’s disease: revising the NINCDS–ADRDA criteria. *Lan. Neurol.* 6 734–746. 10.1016/S1474-4422(07)70178-3 17616482

[B23] Erickson K. I. Boot W. R. Basak C. Neider M. B. Prakash R. S. Voss M. W. (2010). Striatal volume predicts level of video game skill acquisition. *Cereb. Cortex* 20 2522–2530. 10.1093/cercor/bhp293 20089946 PMC3841463

[B24] Erickson K. I. Colcombe S. J. Wadhwa R. Bherer L. Peterson M. S. Scalf P. E. (2007). Training-induced plasticity in older adults: effects of training on hemispheric asymmetry. *Neurobiol. Aging* 28 272–283. 10.1016/j.neurobiolaging.2005.12.012 16480789

[B25] Erickson K. I. Weinstein A. M. Lopez O. L. (2012). Physical activity, brain plasticity, and Alzheimer’s Disease. *Arch. Med. Res.* 43 615–621. 10.1016/j.arcmed.2012.09.008 23085449 PMC3567914

[B26] Folstein M. F. Folstein S. E. McHugh P. R. (1975). Mini-mental state – practical method for grading cognitive state of patients for clinician. *J. Psychiatr. Res.* 12 189–198. 10.1016/0022-3956(75)90026-6 1202204

[B27] Folstein M. F. Folstein S. E. White T. Messer M. A. (2010). *Mini-Mental State Examination*, 2nd Edn Lutz: Psychological Assessment Resources, Inc.

[B28] Friedman N. P. Miyake A. Corley R. P. Young S. E. DeFries J. C. Hewitt J. K. (2006). Not all executive functions are related to intelligence. *Psychol. Sci.* 17 172–179. 10.1111/j.1467-9280.2006.01681.x 16466426

[B29] Garavan H. (1998). Serial attention within working memory. *Mem. Cogn.* 26 263–276. 10.3758/bf03201138 9584434

[B30] Gopher D. Weil M. Siegel D. (1989). Practice under changing priorities – an approach to the training of complex skills. *Acta Psychol.* 71 147–177. 10.1016/0001-6918(89)90007-3

[B31] Heinzel S. Schulte S. Onken J. Duong Q. L. Riemer T. G. Heinz A. (2014). Working memory training improvements and gains in non-trained cognitive tasks in young and older adults. *Aging Neuropsychol. Cogn.* 21 146–173. 10.1080/13825585.2013.790338 23639070

[B32] Jaeggi S. M. Buschkuehl M. Jonides J. Perrig W. J. (2008). Improving fluid intelligence with training on working memory. *Proc. Natl. Acad. Sci. U.S.A.* 105 6829–6833. 10.1073/pnas.0801268105 18443283 PMC2383929

[B33] Jaeggi S. M. Studer-Luethi B. Buschkuehl M. Su Y.-F. Jonides J. Perrig W. J. (2010). The relationship between N-back performance and matrix reasoning—implications for training and transfer. *Intelligence* 38 625–635. 10.1016/j.intell.2010.09.001

[B34] Karbach J. Verhaeghen P. (2014). Making working memory work: a meta-analysis of executive-control and working memory training in older adults. *Psychol. Sci.* 25 2027–2037. 10.1177/0956797614548725 25298292 PMC4381540

[B35] Knoke J. D. (1991). Nonparametric analysis of covariance for comparing change in randomized studies with baseline values subject to error. *Biometrics* 47 523–533. 10.2307/2532143 1912259

[B36] Kramer A. F. Hahn S. Gopher D. (1999a). Task coordination and aging: explorations of executive control processes in the task switching paradigm. *Acta Psychol.* 101 339–378. 10.1016/s0001-6918(99)00011-6 10344190

[B37] Kramer A. F. Larish J. F. Strayer D. L. (1995). Training for attentional control in dual-task settings – a comparison of young and old adults. *J. Exp. Psychol. Appl.* 1 50–76. 10.1037/1076-898x.1.1.50

[B38] Kramer A. F. Larish J. L. Weber T. A. Bardell L. (1999b). “Training for executive control: task coordination strategies and aging,” in *Attention and Performance XVIII: Cognitive Regulation of Performance: Interaction of Theory and Application* Vol. 17 eds Gopher D. Koriats A. (Cambridge, MA: MIT Press), 617–652.

[B39] Kyllonen P. C. Christal R. E. (1990). Reasoning ability is (little more than) working-memory capacity. *Intelligence* 14 389–433. 10.1016/s0160-2896(05)80012-1

[B40] Lee H. Boot W. R. Basak C. Voss M. W. Prakash R. S. Neider M. (2012a). Performance gains from directed training do not transfer to untrained tasks. *Acta Psychol.* 139 146–158. 10.1016/j.actpsy.2011.11.003 22133724

[B41] Lee H. Voss M. W. Prakash R. S. Boot W. R. Vo L. T. K. Basak C. (2012b). Videogame training strategy- induced change in brain function during a complex visuomotor task. *Behav. Brain Res.* 232 348–357. 10.1016/j.bbr.2012.03.043 22504276

[B42] Lewis K. L. Zelinski E. M. (2010). List and text recall differ in their predictors: replication over samples and time. *J. Gerontol. Ser. B Psychol. Sci. Soc. Sci.* 65 449–458. 10.1093/geronb/gbq034 20498454 PMC2949299

[B43] Love J. Selker R. Marsman M. Jamil T. Dropmann D. Verhagen A. J. (2015). *JASP (Version 0.7.1)[Computer Software].*

[B44] Mayr U. Kliegl R. Krampe R. T. (1996). Sequential and coordinative processing dynamics in figural transformations across the life span. *Cognition* 59 61–90. 10.1016/0010-0277(95)00689-3 8857471

[B45] McElree B. (2001). Working memory and focal attention. *J. Exp. Psychol. Learn. Mem. Cogn.* 27 817–835. 10.1037/0278-7393.27.3.817 11394682 PMC3077110

[B46] Melby-Lervåg M. Hulme C. (2013). Is working memory training effective? A meta-analytic review. *Dev. Psychol.* 49 270–291. 10.1037/a0028228 22612437

[B47] Morey R. D. Rouder J. N. (2015). *Bayes Factor (Version 0.9.11-3)[Computer software].*

[B48] Oberauer K. (2002). Access to information in working memory: exploring the focus of attention. *J. Exp. Psychol. Learn. Mem. Cogn.* 28 411–421. 10.1037/0278-7393.28.3.411 12018494

[B49] Oberauer K. (2006). Is the focus of attention in working memory expanded through practice? *J. Exp. Psychol. Learn. Mem. Cogn.* 32 197–214. 10.1037/0278-7393.32.2.197 16569141

[B50] Open Science Collaboration (2015). Estimating the reproducibility of psychological science. *Science* 349 1–8. 10.1126/science.aac4716 26315443

[B51] Park D. C. Bischof G. N. (2010). “Neuroplasticity, aging, and cognitive function,” in *Handbook of the Psychology of Aging*, eds Schaie K. W. Willis S. L. (Cambridge, MA: Academic Press).

[B52] Park D. C. Lodi-Smith J. Drew L. Haber S. Hebrank A. Bischof G. N. (2014). The impact of sustained engagement on cognitive function in older adults: the synapse project. *Psychol. Sci.* 25 103–112. 10.1177/0956797613499592 24214244 PMC4154531

[B53] Park D. C. Reuter-Lorenz P. (2009). The adaptive brain: aging and neurocognitive scaffolding. *Annu. Rev. Psychol.* 60 173–196. 10.1146/annurev.psych.59.103006.093656 19035823 PMC3359129

[B54] Prakash R. S. De Leon A. A. Mourany L. Lee H. Voss M. W. Boot W. R. (2012). Examining neural correlates of skill acquisition in a complex videogame training program. *Front. Hum. Neurosci.* 6:11 10.3389/fnhum.2012.00115 PMC3351675 22615690

[B55] Raven J. C. (1942). Testing the mental ability of adults. *Lancet* 239 115–117. 10.1016/S0140-6736(00)79361-5

[B56] Raz N. Ghisletta P. Rodrigue K. M. Kennedy K. M. Lindenberger U. (2010). Trajectories of brain aging in middle-aged and older adults: regional and individual differences. *Neuroimage* 51 501–511. 10.1016/j.neuroimage.2010.03.020 20298790 PMC2879584

[B57] Redick T. S. Shipstead Z. Harrison T. L. Hicks K. L. Fried D. E. Hambrick D. Z. (2013). No evidence of intelligence improvement after working memory training: a randomized, placebo-controlled study. *J. Exp. Psychol. Gen.* 142 359–379. 10.1037/a0029082 22708717

[B58] Rouder J. N. Morey R. D. Speckman P. L. Province J. M. (2012). Default bayes factors for ANOVA designs. *J. Math. Psychol.* 56 356–374. 10.1016/j.jmp.2012.08.001

[B59] Stepankova H. Lukavsky J. Buschkuehl M. Kopecek M. Ripova D. Jaeggi S. M. (2014). The malleability of working memory and visuospatial skills: a randomized controlled study in older adults. *Dev. Psychol.* 50 1049–1059. 10.1037/a0034913 24219314

[B60] Stine-Morrow E. A. L. Basak C. (2011). “Cognitive interventions,” in *Handbook of the Psychology of Aging*, 7th Edn, eds Schaie K. W. Willis S. L. (New York, NY: Elsevier), 153–171.

[B61] Strobach T. Frensch P. Müller H. Schubert T. (2015). Evidence for the acquisition of dual-task coordination skills in older adults. *Acta Psychol.* 160 104–116. 10.1016/j.actpsy.2015.07.006 26231939

[B62] Tabachnick G. G. Fidell L. S. (2007). *Experimental Designs Using ANOVA.* Belmont, CA: Duxbury.

[B63] Vaughan L. Basak C. Hartman M. Verhaeghen P. (2008). Aging and working memory inside and outside the focus of attention: dissociations of availability and accessibility. *Neuropsychol. Dev. Cogn. Sec. B Aging Neuropsychol. Cogn.* 15 703–724. 10.1080/13825580802061645 18608047

[B64] Verhaeghen P. Basak C. (2005). Ageing and switching of the focus of attention in working memory: results from a modified N-Back task. *Q. J. Exp. Psychol. Sec. Hum. Exp. Psychol.* 58 134–154. 10.1080/02724980443000241 15881295

[B65] Verhaeghen P. Cerella J. Basak C. (2004). A working memory workout: how to expand the focus of serial attention from one to four items in 10 hours or less. *J. Exp. Psychol. Learn. Mem. Cogn.* 30 1322–1337. 10.1037/0278-7393.30.6.1322 15521807

[B66] Verhaeghen P. Cerella J. Bopp K. L. Basak C. (2005). “Aging and varieties of cognitive control: a review of meta-analyses on resistance to interference, coordination and task switching, and an experimental exploration of age-sensitivity in the newly identified process of focus switching,” in *Cognitive Limitations in Aging and Psychopathology: Attention, Working Memory, and Executive Functions*, eds Engle R. W. Sedek G. von Hecker U. McIntosh D. N. (Cambridge, MA: Cambridge University Press), 160–179.

[B67] Verhaeghen P. Salthouse T. A. (1997). Meta-analyses of age-cognition relations in adulthood: estimates of linear and nonlinear age effects and structural models. *Psychol. Bull.* 122 231–249. 10.1037/0033-2909.122.3.231 9354147

[B68] Voss M. W. Prakash R. S. Erickson K. I. Boot W. R. Basak C. Neider M. B. (2012). Effects of training strategies implemented in a complex videogame on functional connectivity of attentional networks. *Neuroimage* 59 138–148. 10.1016/j.neuroimage.2011.03.052 21440644

[B69] Wechsler D. (1939). *The Measurement of Adult Intelligence.* Baltimore, MD: Williams & Wilkins.

[B70] Zinke K. Zeintl M. Eschen A. Herzog C. Kliegel M. (2012). Potentials and limits of plasticity induced by working memory training in old-old age. *Gerontology* 58 79–87. 10.1159/000324240 21430358

[B71] Zinke K. Zeintl M. Rose N. S. Putzmann J. Pydde A. Kliegel M. (2014). Working memory training and transfer in older adults: effects of age, baseline performance, and training gains. *Dev. Psychol.* 50 304–315. 10.1037/a0032982 23688173

