# Supplementary material for: To Switch or Not to Switch: Role of Cognitive Control in Working Memory Training in Older Adults
Source: Front Psychol. 2016 Mar 2;7:230. doi: 10.3389/fpsyg.2016.00230 (PMC4774648; doi:10.3389/fpsyg.2016.00230)
Supplement: Supplementary file 1 [file Table_1.PDF]

Supplementary Table 1

Number of participants (N), Means (M) and Standard Deviations (SD) for the transfer tasks at baseline, immediate post, and delayed post for both PT and UT.

		Baseline		Immediate Post		Delayed Post	
Transfer Tasks		PT	UT	PT	UT	PT	UT
DSST	*N*	21	22	21	22	14	17
	M(SD)	16.52(4.37)	16.18(3.65)	16.90(3.10)	16.91(4.79)	17.93(3.93)	17.12(3.87)
SingleRT	*N*	21	22	21	22	14	17
	M(SD)	780.08(120.07)	787067(146.67)	742.14(116.94)	779.34(133.95)	806.29(133.85)	761.62(130.84)
ForwardSpan	*N*	21	22	21	22	14	17
	M(SD)	7.14(1.11)	6.82(1.14)	7.10(1.18)	6.95(1.36)	7.07(1.21)	7.24(0.90)
BackwardSpan	*N*	21	22	21	22	14	17
	M(SD)	4.71(1.31)	4.77(0.87)	4.76(1.09)	5.14(1.04)	5.00(1.18)	5.65(1.66)
DualSwitchCost	*N*	21	22	21	22	14	17
	M(SD)	223.14(174.90)	227.98(143.57)	189.54(149.61)	239.05(168.83)	221.53(136.74)	298.59(125.25)
UnpredSwitchCost	*N*	21	22	21	22	14	17
	M(SD)	233.31(174.71)	242.41(133.95)	194.28(176.79)	255.53(189.80)	242.36(150.49)	259.21(157.92)
RAPM	*N*	21	22	21	22	14	17
	M(SD)	6.86(3.50)	6.59(3.19)	6.19(3.19)	6.95(3.21)	8.07(3.29)	6.53(2.81)
StoryRecall	*N*	21	22	21	22	14	17
	M(SD)	22.14(3.21)	20.18(5.28)	21.95(3.37)	22.77(4.42)	18.57(4.03)	18.59(3.22)
MMSE	*N*	21	22	21	22	14	17
	M(SD)	29.00(1.30)	28.68(1.67)	28.81(1.69)	29.14(1.52)	28.43(1.79)	28.12(2.42)
