# Supplementary material for: To Switch or Not to Switch: Role of Cognitive Control in Working Memory Training in Older Adults
Source: Front Psychol. 2016 Mar 2;7:230. doi: 10.3389/fpsyg.2016.00230 (PMC4774648; doi:10.3389/fpsyg.2016.00230)
Supplement: Supplementary file 2 [file Table_2.PDF]

Supplementary Table 2

ANCOVA was conducted on each transfer task to evaluate any differences between the two training groups (PT vs. UT) at both immediate post testing as well as delayed post testing, after controlling for the individual differences in the baseline score and inFoA lng.

Variables	Immediate Post	Retention
DSST	$F(1, 39)=0.01$, $p=.92$, $MSE=14.23$, $\eta^2_P=0$	$F(1, 27)=0.27$, $p=.61$, $MSE=7.05$, $\eta^2_P=.01$
SingleRT	$F(1, 38)=1.12$, $p=.30$, $MSE=13308.39$, $\eta^2_P=.03$	$F(1, 26)=0.23$, $p=.64$, $MSE=16139.62$, $\eta^2_P=.01$
ForwardSpan	$F(1, 39)=0.32$, $p=.57$, $MSE=0.99$, $\eta^2_P=.01$	$F(1, 27)=0.98$, $p=.33$, $MSE=0.93$, $\eta^2_P=.04$
BackwardSpan	$F(1, 39)=1.28$, $p=.27$, $MSE=0.91$, $\eta^2_P=.03$	$F(1, 27)=0.86$, $p=.36$, $MSE=2.19$, $\eta^2_P=.03$
DualSwitchCost	$F(1, 38)=2.06$, $p=.16$, $MSE=14734.09$, $\eta^2_P=.05$	$F(1, 26)=1.71$, $p=.20$, $MSE=11639.77$, $\eta^2_P=.06$
UnpredFocusSwitch	$F(1, 38)=1.34$, $p=.26$, $MSE=22480.48$, $\eta^2_P=.03$	$F(1, 26)=0.01$, $p=.92$, $MSE=18243.35$, $\eta^2_P=0$
RAPM	$F(1, 39)=2.73$, $p=.11$, $MSE=4.27$, $\eta^2_P=.07$	$F(1, 27)=1.34$, $p=.26$, $MSE=4.89$, $\eta^2_P=.05$
StoryRecall	$F(1, 39)=3.09$, $p=0.09$, $MSE=11.04$, $\eta^2_P=.07$	$F(1, 27)=0.04$, $p=.84$, $MSE=13.47$, $\eta^2_P=0$
