# Supplementary material for: To Switch or Not to Switch: Role of Cognitive Control in Working Memory Training in Older Adults
Source: Front Psychol. 2016 Mar 2;7:230. doi: 10.3389/fpsyg.2016.00230 (PMC4774648; doi:10.3389/fpsyg.2016.00230)
Supplement: Supplementary file 3 [file Data_Sheet_1.PDF]

Supplementary Materials

Baseline Comparisons of the Transfer Tasks

The two groups of randomly assigned older adults (PT vs. UT) did not vary at baseline in any measures of the transfer tasks, viz., DSST, $t(41)$=0 .09, p=.93, SingleRT, $t(40)$=0.01, p=.99, ForwardSpan, $t(41)$= 0.94, p=.35, BackwardSpan, $t(41)$= -0.22, p=.83, DualSwitchCost, $t(40)$= 0.07, p=.95, UnpredSwitchCost, $t(40)$= -0.09, p=.93, RAPM, $t(41)$= 0.20, p=.85, and StoryRecall, $t(41)$= 1.16, p=.25.

Learning-related Changes in Accuracy of the Trained Tasks

To evaluate whether the accurate performance in and out of the FoA differed across training group across the 5 hr of training, we conducted a 2 (Training_type) x 2 (FoA: inFoA and outFoA) x 5 (Day) repeated measures ANOVA. The main effect for FoA was significant, F(1, 40)=167.12, p<.001, MSE=.03, η^2_p=.81, with items inside the FoA having greater accuracy than items outside the FoA. The main effect of Day was also significant, F(1, 160)=4.09, p=.04, MSE=.02, η^2_p=.09. The post-hoc repeated contrasts found that each day had significantly greater accuracy than the prior day. No other variables or interactions were significant. That is, PT and UT groups did not differ in their performance either inside or outside the FoA, nor across the 5 hr of training (Supplementary Figure 1b).

Figure Captions

Supplementary Figure 1. (a) Learning rates and (b) average accuracy for information units both inside and outside the focus of attention did not differ between the two training groups, PT and UT.

Note: inFoA PT = inside the FoA for the predictable training group, inFoA UT = inside the FoA for the unpredictable training group, outFoA PT = outside the FoA for the predictable training group, outFoA UT = outside the FoA for the unpredictable training group.
