# Supplementary figures and images for: To Switch or Not to Switch: Role of Cognitive Control in Working Memory Training in Older Adults
Source: Front Psychol. 2016 Mar 2;7:230. doi: 10.3389/fpsyg.2016.00230 (PMC4774648; doi:10.3389/fpsyg.2016.00230)

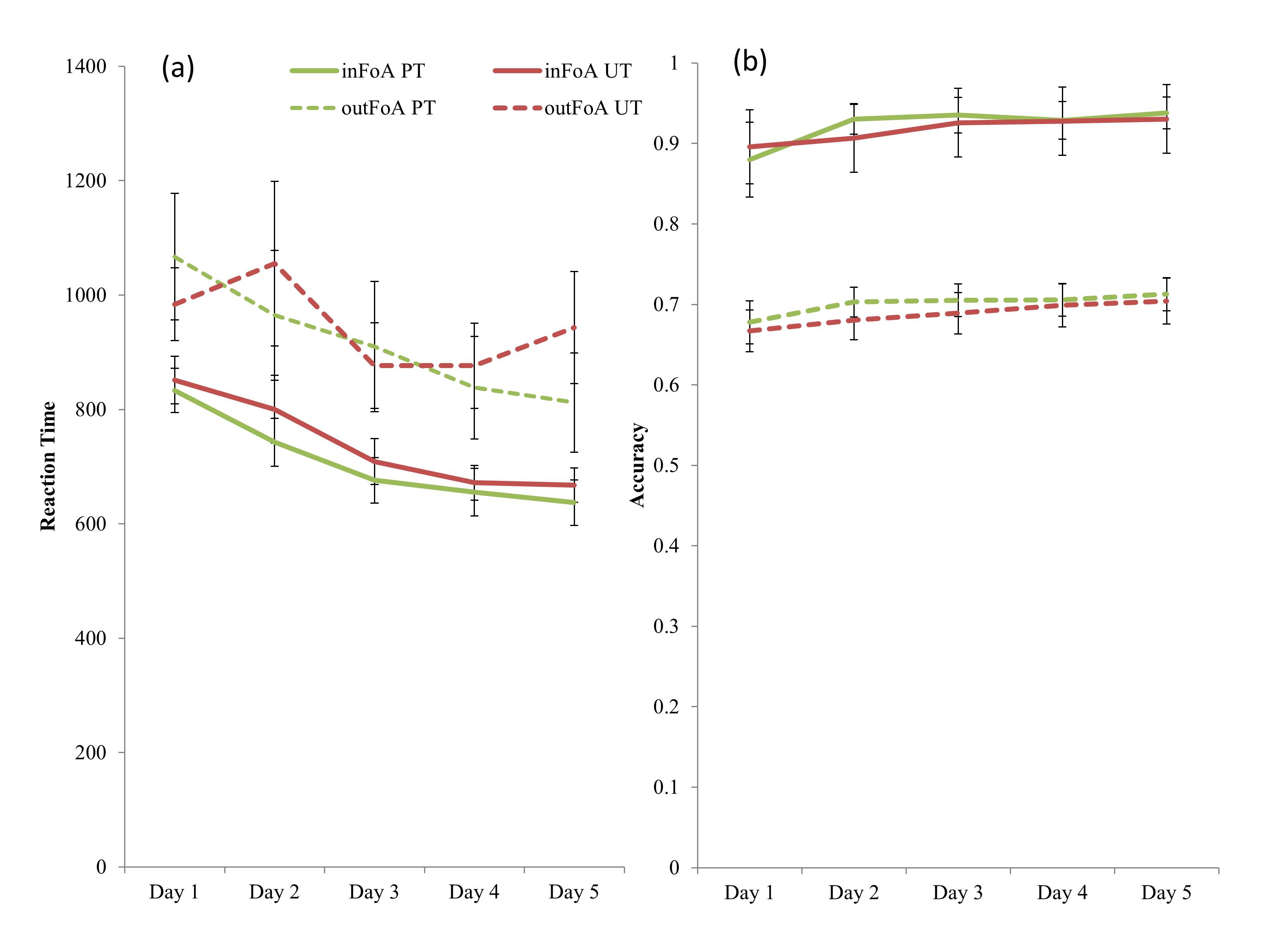

Supplement: Supplementary file 4 [file Image_1.JPEG]
